# Dynamics of Adaptive Alleles in Divergently Selected Body Weight Lines of Chickens

**DOI:** 10.1534/g3.113.008375

**Published:** 2013-10-29

**Authors:** Mats E. Pettersson, Anna M. Johansson, Paul B. Siegel, Örjan Carlborg

**Affiliations:** * Department of Clinical Sciences, Swedish University of Agricultural Sciences, 750 07, Uppsala, Sweden; † Department of Animal Breeding and Genetics, Swedish University of Agricultural Sciences, 750 07, Uppsala, Sweden; ‡ Department of Animal and Poultry Sciences, Virginia Polytechnic Institute and State University, Blacksburg, Virginia 24061-0306

**Keywords:** divergent selection, body weight, chicken, artificial selection, selective sweeps

## Abstract

By studying genomic changes over time in populations subjected to strong artificial directional selection, we can gain insights to the dynamics of beneficial alleles originating from the founder population or emerging as novel mutations undergoing ongoing selection. The Virginia lines are a chicken resource population generated by long-term bi-directional, single-trait selection for juvenile body weight. We studied genome-wide allele frequency changes from generation 40 to 53 using genome-wide genotypes from directional and relaxed selection lines. Overall, there were small changes in allele frequencies at individual loci over the studied time period; but, on average, the changes were greater in lines with larger phenotypic changes. This is consistent with previous findings that much of the response to selection over the first 40 years of selection was attributable to utilization of standing genetic variation at many loci in the genome, indicating a mostly polygenic architecture for body weight. Over the course of the selection experiment, the largest phenotypic response to selection was observed in the high-weight selected line, and in this line we detected a single locus where the allele frequency changed rapidly during a late stage of the experiment. This locus likely contains a novel, beneficial mutation that appeared between generations 40 and 45 and was driven to fixation in 5 to 10 generations. This result illustrates the dependence of continued long-term selection response on standing genetic variation at many loci as well as strong, novel, beneficial mutations.

Evolution can be described as genomic change driven by selection for phenotypes with adaptive advantages. The basic concept is simple, but unraveling the details of the process is far from straightforward. A poorly understood, but central, topic for our understanding of adaption is the origin and fate of beneficial mutations (Orr 2010). Although beneficial alleles might be difficult to identify and trace in natural populations, artificial selection experiments can be designed to explore the dynamic processes in the genome involving such alleles. For decades, artificial selection experiments have been used in quantitative genetics to empirically evaluate theoretical predictions about selection responses. Examples include selection from inbred base populations to test the contribution of mutational variance in quantitative traits and prediction of selection response from genetic variation present in the base population (Hill and Caballero 1992). Artificially selected populations can be subjected to much stronger selection pressures than natural populations, thus their adaption to a new experimental environment can be considered to be a process of accelerated evolution. This has also been utilized in several recent studies in which data from existing and new long-term selection experiments have been used to provide empirical insights to the genome-wide distribution and dynamics of adaptive mutations (Burke 2012; Johansson *et al.* 2010). By searching for selective sweeps (Berry *et al.* 1991) in the selected populations, *i.e.*, regions of the genome that display little variation, it is possible to identify regions that are likely to contain alleles that are strongly selected (Maynard Smith and Haigh 1974). The patterns of fixation will vary depending on whether selection has acted on new mutations (leading to so called hard sweeps) or standing genetic variation (leading to so-called soft sweeps). Such studies have been performed in both selected sexual and asexual populations and have provided insights to adaption from beneficial alleles originating in the standing variation in the base population as well as appearing as new mutations during selection. One of the currently outstanding questions involves the relative frequency of adaption from the two in a single population, because this has not yet been empirically tested in a single experimental system (Burke 2012).

The Virginia lines are experimental populations founded in 1957 by intercrossing seven partially inbred lines of White Plymouth Rock chickens and, since then, has been extensively used for studying the genetic, genomic, and phenotypic effects of long-term, single trait, divergent artificial selection (Dunnington and Siegel 1996). Once per year, the birds with the highest and lowest 8-week body weights, with some restrictions imposed to minimize inbreeding, within each line are selected as parents for the next generation (Márquez *et al.* 2010). Currently, after 53 generations of selection, there is a 12-fold difference in body weight between the lines.

By searching for soft sweeps in the Virginia lines, we showed that approximately 100 genomic regions have been fixed for alternative alleles in the high-weight and low-weight selected lines after 50 generations of selection (Johansson *et al.* 2010). This indicated that standing genetic variation at many loci present in the founder population made a significant contribution to the realized selection response in these lines. Although we cannot exclude that some of the detected sweeps are attributable to novel mutations, it is unlikely because they were inferred by their fixation for alternative alleles in the two divergently selected populations. This pattern of fixation is expected to be rare unless both alleles were present in the base population. In this article, we have extended the previous work by performing in-depth analyses to detect selective sweeps that are novel within the high-weight and low-weight selected lines and therefore are more likely to be attributable to novel mutations. By also including later generations of selected birds, as well as two derivative relaxed selection lines founded by the high-weight and low-weight lines at generation 45, the analysis not only facilitates detection of novel sweeps on mutations that occurred after the divergence of the lines but also allows for estimation of when the mutation occurred. Birds from the high-weight selected (HWS) and low-weight selected (LWS) lines were sampled at generations 40, 50 (Johansson *et al.* 2010), and 53 and individually genotyped using a 60-k SNP chip. In addition, we sampled birds from generation nine of two relaxed lines (high-weight relaxed [HWR] and low-weight relaxed [LWR]) that were founded by generation 45 of HWS and LWS, respectively. A schematic describing the relationship between the studied groups is provided in Figure 1.

**Figure 1:**
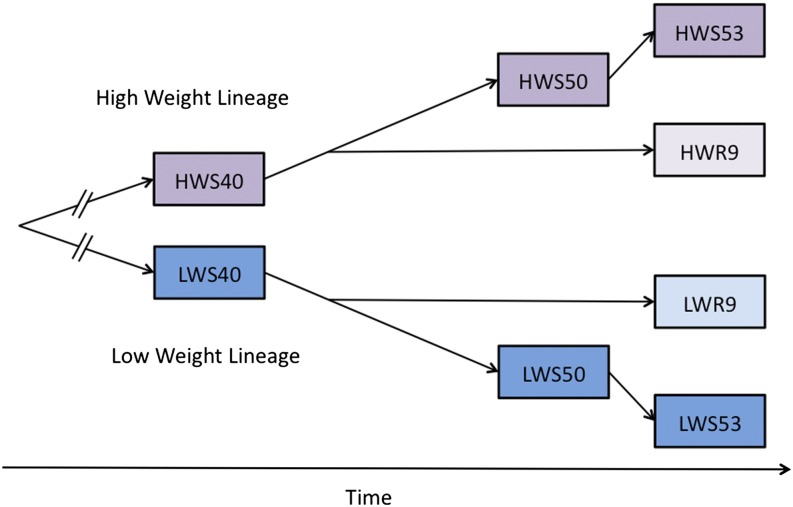
Schematic of the genotyped populations. An illustration of the relationship between, and the number of genotyped individuals within, each sampled sub-line and generation from the Virginia chicken lines.

Here, we report the results from analyses of the changes of allele frequencies over time, scans for stretches of fixed, or nearly fixed, markers, LD block analyses, and studies of divergence over time between the related subgroups to provide a general overview of the genome dynamics in these lines. The results indicate a general trend of small allele frequency changes across a large number of loci, supporting the findings from our previous studies that much of the selection response is attributable to selection on standing variation at many genomic loci and that the genetic architecture of body weight is highly polygenic (Johansson *et al.* 2010; Jacobsson *et al.* 2005). However, we were also able to identify strong recent selection at an individual locus in the HWS lineage, which is likely the result of a beneficial, novel mutation appearing late in the selection experiment. Thus, the results reported here support the general consensus that the genetic architecture of body size mainly comprises a large number of genes with intermediate to small individual effects with contributions from a small number of loci with larger effects (Hu *et al.* 2007; Lango Allen *et al.* 2010; Visscher *et al.* 2010). Furthermore, they also question the validity of the conclusions of previous studies involving dogs (Sutter *et al.* 2007; Boyko *et al.* 2010) and horses (Makvandi-Nejad *et al.* 2012) that the genetics of stature in these species is primarily influenced by variation in one or a few major genes and suggests that further studies are needed before it can be concluded that those species have a different genetic architecture underlying these traits.

## Materials and Methods

### Ethics statement

All procedures involving animals used in this experiment were performed in accordance with the Virginia Tech Animal Care Committee animal use protocols.

### Birds and genotyping

Genotyping was performed on 20 chickens, per group, from generation 53 of the HWS and LWS Virginia lines as well as from generation 9 of the relaxed lines derived from the HWS and LWS lines at generation 45. In addition to this, we also used the genotypes from HWS and LWS lines at generations 40 and 50 that were used by Johansson *et al.* (2010) (Figure 1). DNA Landmarks performed the genotyping using the 60-K chicken chip produced by Illumina for the GWMAS Consortium. After quality control, in which markers with 10 or more missing genotypes were excluded, 55,983 makers were retained in the analysis (Genotypes and metadata are available in File S5). The animal husbandry was consistent throughout the selection experiment, as has been previously described in detail (Dunnington and Siegel 1996; Márquez *et al.* 2010).

### Detection of selective sweeps

Selective sweeps are detected by screening the genome for regions of low within-population variability. We measured this variability by the expected heterozygosity, calculated as in Equation 1.

$$H_{z}=1-\left(m^{2}+{\left(1-m\right)}^{2}\right),\;\text{where}\;m\;\text{is}\;\text{the}\;\text{major}\;\text{allele}\;\text{frequency}.$$ (1)

This value was first calculated per marker and then used to estimate moving averages over a window of consecutive markers, which is the measure used to infer selective sweeps in our analysis. The averaging is important as a sweep and, by definition, spans a region that is expected to have a consistent signal. Unless averaging is performed, the analyses will be affected by noise from individual fixed markers that do not necessarily indicate a selective sweep but rather could be attributable to, for example, uneven allele frequencies across haplotypes in the founding population leading to early fixation for alternative alleles in the selected lines by drift. This study covers 13 generations, indicating that there will be a significant linkage drag around selected loci in this study. We therefore evaluated a realistic range of window sizes (from 20–500 markers, corresponding to ≈2–30 cM), given the length of the chicken genetic map (3800 cM) (Groenen *et al.* 2000) and our previous work regarding the distribution of lengths of selective sweeps in this population (Johansson *et al.* 2010). The choice of window length had some quantitative effects on the results, but no major impact on the qualitative outcome. Based on these evaluations, we therefore used a window size of 100 markers throughout this study, because it was the window size that best matched the observed “wavelength” of fluctuations in heterozygosity along the genome. The within-population heterozygosity profiles for the different subgroups (Figure 1) were compared in a pair-wise manner in accordance with the experimental design to separate signals of selection and drift, as described by Johansson *et al.* (2010). By combining the results of the within-lineage and between-lineage (that is, HWS or LWS) pairwise comparisons with those obtained from comparisons between sublines (HWS *vs.* HWR and LWS *vs.* LRW), we can establish a timeline of fixations events based on the subgroups within which a given fixation is present.

### Estimating the significance of heterozygosity changes

We used the observed window-wise distribution of changes in heterozygosity across the genome to empirically estimate the deviation induced by genetic drift. The assumption is that most of the regions in the genome are under no, or weak, selection during the time-span evaluated in this study and, consequently, nearly all the observed differences in the evaluated windows are attributable to genetic drift. This is, in practice, an overestimate because some loci will be undergoing selection and lead to larger changes in allele frequencies than can be explained by drift only. Thus, a window that, after multiple testing corrections, is identified as a significant outlier in this distribution is likely to have been subject to selection. The threshold used to identify the outliers was set at the 4.2*10^−5^ quantile of a normal distribution with the same mean and SD as the observed empirical distribution, corresponding to a 0.025 threshold (*i.e.*, a 5% two-sided test) Bonferroni-corrected for the 560 nonoverlapping windows of 100 markers needed to cover the genome.

### Test for allele frequency differences between populations

To test whether there was a significant global change in allele frequencies between time points and thus a divergence over the separating time interval between the lines, we applied the Fisher exact test for differences in allele frequency between the relaxed and their ancestral selected lines (HWS40 and LWS40, respectively) at all markers. The threshold used (8.9*10^−7^) corresponds to a threshold of 0.05 Bonferroni-corrected for the number of markers tested (55,983). This is rather conservative because linkage disequilibrium among close markers reduces the effective number of comparisons.

### Statistical analysis software

The statistical analysis was performed using custom written code in R (R Development Core Team 2011). The R code is provided in the Supporting Information, File S4.

### Haplotype analysis

The program Haploview (Barrett *et al.* 2005) was used to analyze haplotype blocks separately in generations 40 and 50 in each selection line. SNP pairs closer than 10 Mb were used for pairwise comparisons and the haplotype block definition of Gabriel *et al.* (2002) was used. The output was then summarized and compared between populations and chromosomes using custom-made R-scripts.

## Results

### Age of fixation events

**Figure 2:**
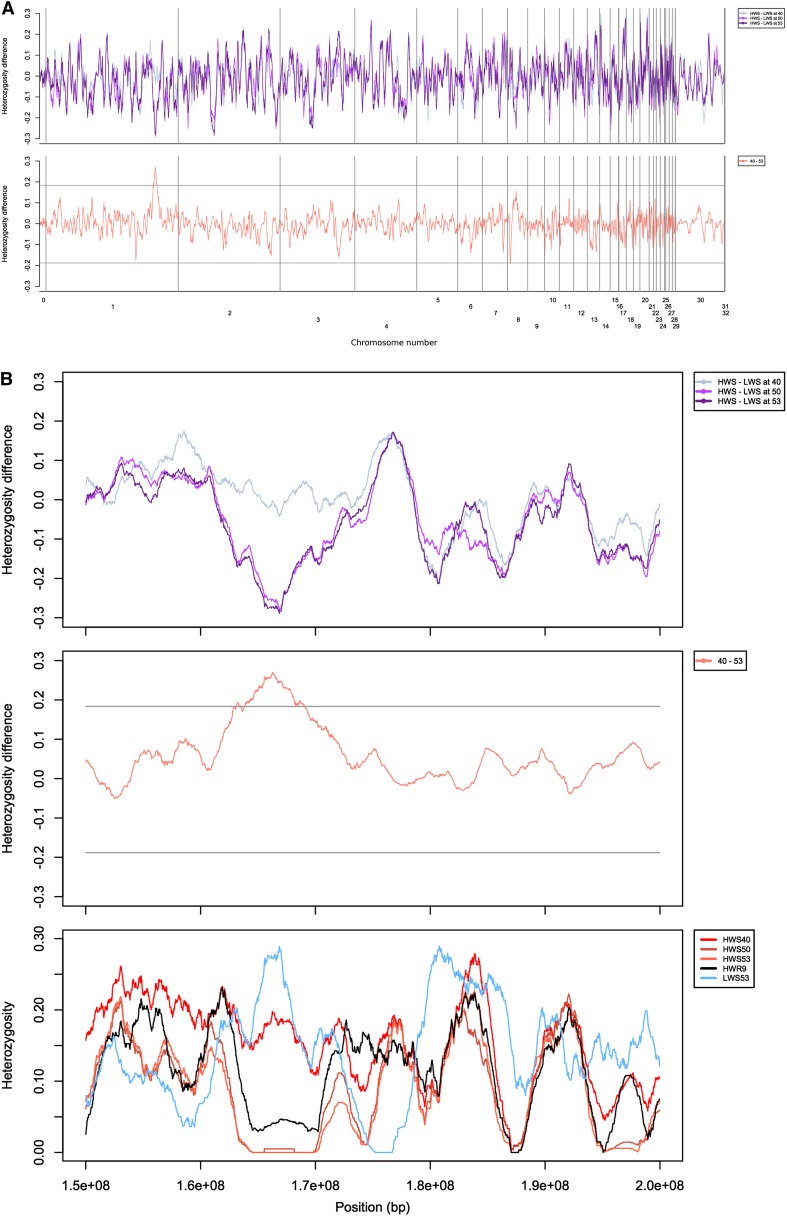
Genome-wide heterozygosity profiles. (A) Top panel shows heterozygosity differences for the pairs HWS and LWS at generations 40, 50, and 53; bottom panel shows the change in these profiles between generations 40 and 53. Regions showing significant changes in allele frequencies are indicated by their crossing the multiple testing corrected global two-sided 0.025 significance threshold. (B) Top panel presents zoom-in on the region on GGA1 that contains a significant change in the heterozygosity difference of the pair HWS and LWS from generation 40 to generation 53; bottom panel shows the absolute heterozygosity level for all eight populations in the GGA1 region. Note that there is low heterozygosity (but not fixation) in the GGA1 region in HWR9.

Figure 2A (top pane) shows the genome-wide profiles for differences in heterozygosity between the HWS and the LWS at the three sampled time points (generations 40, 50, and 53) scored in a sliding window of 100 markers. The lower pane shows the difference between the curves from generations 40 and 53 in the top profile, highlighting the changes that have taken place during these 13 generations. In addition, we have measured the absolute heterozygosity values for all subgroups (File S1, bottom track), which revealed that several large regions, located on GGA1 (approximately 165 Mb), GGA2 (55 Mb and 145 Mb), and GGA4 (75 Mb) are fixed only in the HWS lineage. The lower pane profile illustrating the change between generations 40 and 53 shows that these regions have fixed at different times, because only the region on GGA1 shows evidence of a significant recent change. A chromosome-by-chromosome version of Figure 2A that provides higher resolution is included as File S1, File S2, and File S3.

Figure 2B provides a detailed profile for the region around 165 Mb on GGA1 that displays a large, recent change in allele frequencies in the HWS line. Note that an incomplete version of the sweep in the region on GGA1 is also present in HWR9. As can be seen in the bottom pane of Figure 2B, the exact same region that is fixed in HWS50 has a clear decrease in heterozygosity in HWR9. This illustrates that onset of selection in this region was before generation 45, and that fixation took place later. However, the LWS populations are all similar to each other (data not shown; LWS53 included for reference). Thus, selection in this region occurred only in the HWS, with onset between generation 40, at which time the LWS and HWS were similar, and generation 45 when the HWR line was founded. Also, fixation must have occurred after the splitting-off of HWR. In our opinion, the most likely reason for this sudden strong selection is the occurrence of a strongly beneficial mutation in the HWS lineage.

### Evidence of purifying selection in the relaxed lines

**Figure 3:**
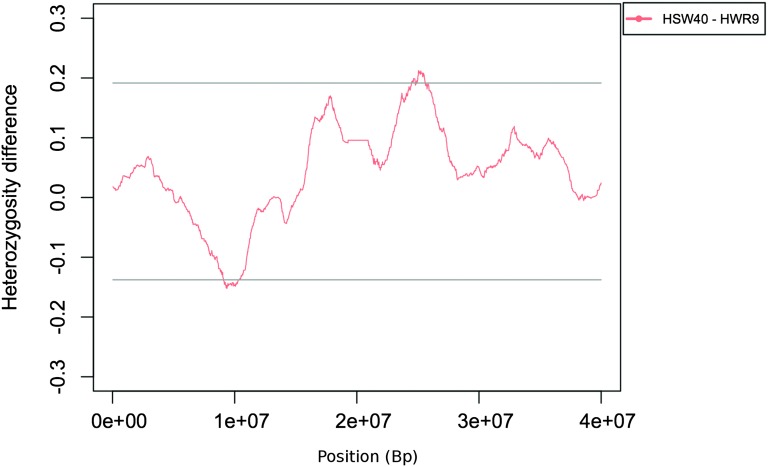
Allele frequency changes on GGA4 in the HWR line lineage relative to HWL40. The figure shows a segment of GGA4 where the HWR9 line has experienced both significant loss and gain in heterozygosity in flanking genome segments in the interval between HWS40 and HWR9. Significant changes in allele frequencies are those that are outside of the multiple testing corrected global two-sided significance threshold.

The overall rate of heterozygosity loss in the genome was higher in HWR9 than in HWS53 and in LWR9 than in LWS53 (Table 1), indicating that there is purging selection acting on previously selected alleles in both relaxed lines. In HWR9, the region displaying the greatest loss is located in the middle of GGA4, shown in Figure 3, which interestingly also has a flanking region where heterozygosity is increasing significantly. Our interpretation of this is that there is ongoing selection on an allele that is in phase with rare marker alleles in that flanking region and that the common marker alleles in the region have gone to fixation. Although this could also be attributable to a novel mutation, it is probably more parsimonious to assume that the recent shift in selection paradigm resulted in purifying selection, as would occur if the selected allele increases juvenile body weight, but at the same time has negative effects on general fitness.

**Table 1:** Genome-wide mean heterozygosity loss over time in the four sub-lines

Population	Mean Heterozygosity Loss
HWS40 to HWS53	−0.018 ± 0.003
HWS40 to HWR9	−0.027 ± 0.004
LWS40 to LWS53	−0.021 ± 0.004
LWS40 to LWR9	−0.026 ± 0.005

**Figure 4:**
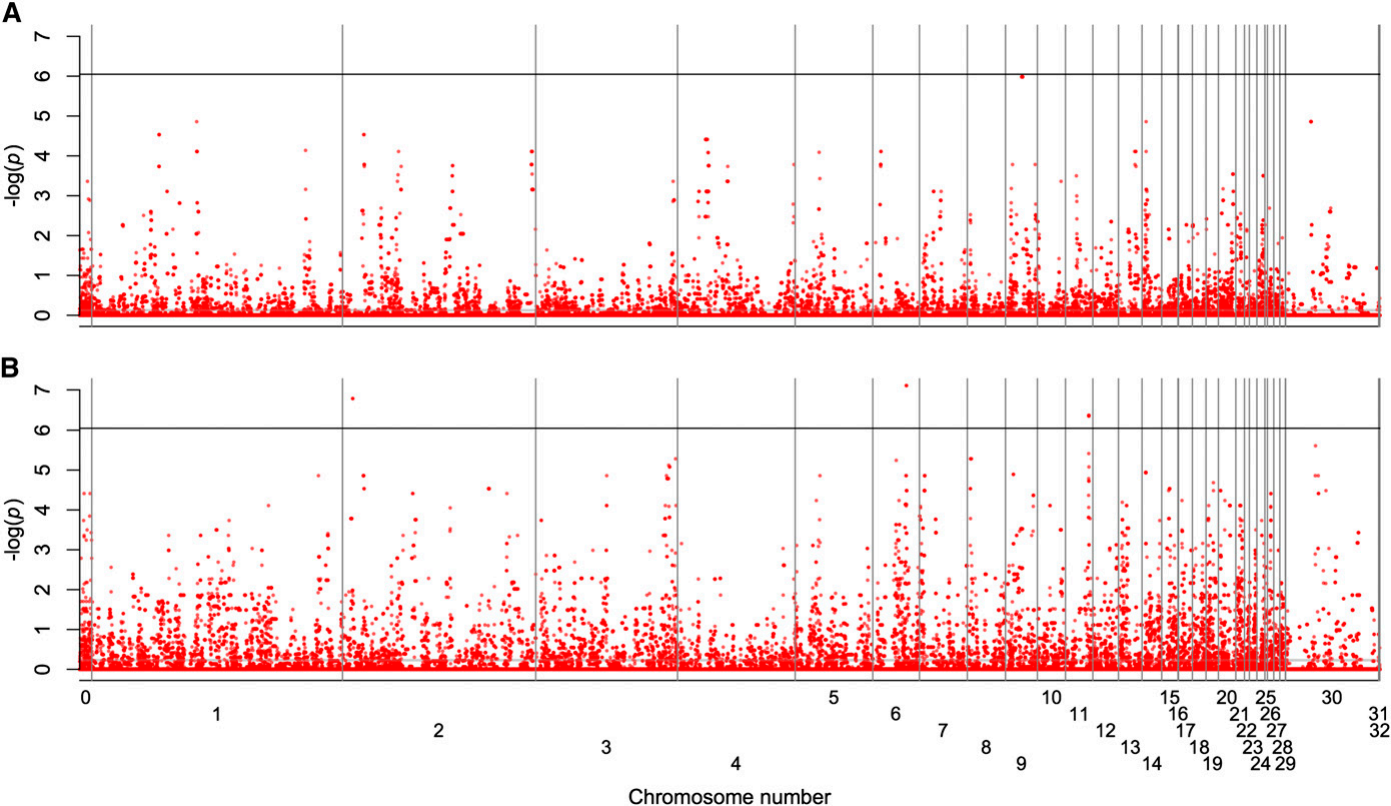
Genome-wide tests for purging selection in HWR and LWR relaxed lines. Results from the Fisher exact test of allele frequency change in the HWS40/HWR and LWS40/LWR pairs to evaluate whether there was a general loss of previously selected alleles in the relaxed lines. Complete genome profiles for the (A) HW and (B) LW pairs, respectively, are shown.

Figure 4 shows the allele frequency difference profiles of the HWS pair (HWS53 and HWR9; Figure 4A) and the LWS pair (LWS53 and LWR9; Figure 4B). Because the selected and relaxed lines share ancestry up to generation 45, the observed differences equal divergence from that point onward. Re-ordering the −log *p*-values for each pair from biggest to smallest and performing a regression between the LWS and HWS pair revealed that the *p*-values in the LWS pair were globally lower (slope, 1.46; SE, 0.0014). Thus, the global amount of difference accumulated between the selected and relaxed lines is greater in the LW lineage. This can be interpreted as a sign of purifying selection, consistent with the observation that the LWS line has reached a phenotypic plateau where further responses to selection are limited by reduced fitness.

### Detection of large LD blocks

**Figure 5:**
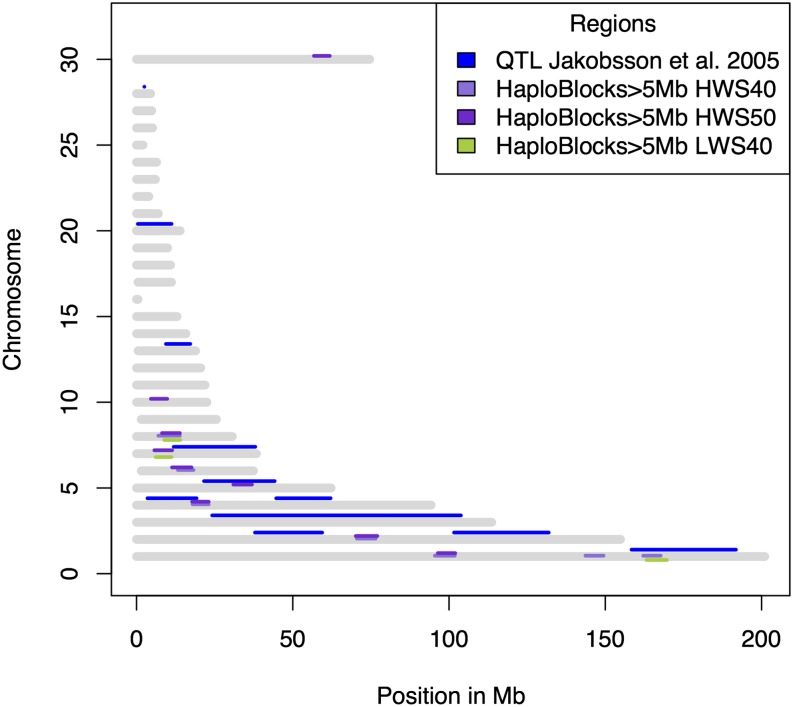
Distribution of large haplotype blocks across the genome of the Virginia chicken lines. The figure shows the location of exceptionally large (>5 Mb) haplotype blocks in the HWS (purple) and LWS (green) lines together with previously identified QTL (blue; 18,19) (Wahlberg *et al.*, 2009; Pettersson *et al.*, 2011).

A number of exceptionally large (>5 Mb) LD blocks were identified in both the HWS and LWS lines using Haploview (Barrett *et al.* 2005). Haplotype blocks larger than 5 Mb were identified in HWS40 (seven blocks), LWS40 (three blocks), and HWS50 (eight blocks), whereas no blocks were found in LWS50, probably because of the limited size of that group. The positions of all blocks are listed in Table 2, and the blocks from generation 40 are graphically displayed in Figure 5. The identified regions largely overlap with previously identified QTL in an intercross between HWS40 and LWS40 (Figure 5). There are three regions with haplotype blocks larger than 5 Mb that occur in both the high line and the low line. These three regions are located on GGA1, GGA7, and GGA8.

**Table 2:** Haplotype blocks in the genome inferred to be larger than 5 Mb

Line*^a^*	Chromosome	Start (Mb)	Stop (Mb)
HWS40	1	95.48	101.85
	1	143.63	149.44
	1	162.14	167.71
	2	70.47	76.51
	4	17.92	23.07
	6	13.22	18.22
	8	6.99	13.85
LWS40	1	163.20	169.73
	7	6.14	11.17
	8	8.89	13.99
HWS50	1	96.42	101.99
	2	70.14	77.04
	4	17.80	23.07
	5	30.99	36.93
	6	11.36	17.59
	7	5.70	11.38
	8	8.14	13.85
	10	4.59	9.79

a Haploblocks could not be detected in LWS50 because of the small sample size.

## Discussion

Long-term artificial selection experiments are useful for experimentally exploring questions of central importance in both quantitative genetics and evolution, including the genome dynamics of beneficial mutations. The nature of single-trait artificial selection—strong and narrow—creates differences compared to natural situations. Specifically, the tolerance for side effects of selected alleles is strongly amplified, because fitness is almost exclusively tied to the selected trait. In terms of unraveling the genetic architecture of the trait, this is an advantage because it removes interference from other systems. However, it also means the actual alleles fixed under the artificial selection regime may have severe drawbacks in natural conditions.

It is known from previous experimental work that both standing genetic variation and novel mutations can contribute to adaption, but little is known about the relative contribution of these two sources of genetic variation. Selection experiments from inbred founders are useful for showing that novel mutations can contribute to selection responses. They do not, however, provide insight regarding how important novel mutations at low frequencies are in relation to existing standing variation. In contrast, selection starting from a genetically variable founder population allows explorations of the realized selection response from standing genetic variation but makes it difficult to assess the impact of novel mutations that may occur, because they cannot easily be detected or monitored.

We analyzed data from a chicken resource population that had been subjected to long-term, single-trait, bi-directional selection for 53 generations. The selection was sufficiently intense to bring the LWS line a selection plateau in approximately 25 generations while there was still a response to selection in the HWS line. By comparing the genomes of the two divergently selected lines using high-density SNP genotypes, we showed that it is likely that a large part of the selection response over the first 40 generations was attributable to selection on standing genetic variation. This led to fixation for alternative alleles in the two lines at approximately 100 loci across the genome (Johansson *et al.* 2010). In the present study, our focus was on studying the genome dynamics separately within each line over the past 13 years of selection in an attempt to identify the most likely source of the genetic variants currently undergoing selection.

### Rapid allele frequency changes in one region on GGA1 in the HWS line

A genomic region spanning from 162 to 170 Mb on GGA1 displays considerable recent loss of variation. In addition, the region also contains very large haplotype blocks and a QTL affecting growth-related traits has been observed in this region (Jacobsson *et al.* 2005; Wahlberg *et al.* 2009). This sweep is present, but incomplete, in HWR9, indicating that at generation 45, when the HWR was splintered-off from the HWS stock, selection had started but fixation had not yet occurred. The size of the region suggests that the onset of selection cannot be far back into the pedigree, because the 8-Mb region is expected to correspond to a linkage distance of approximately 25 cM, using the average physical distance per cM in chicken (Groenen *et al.* 2009); breakdown attributable to recombination would be expected to be very rapid.

A possible explanation for the recent changes in the allele frequencies would be a recently initiated selection on a mutation that was present in the base population but whose effect had increased because of a change in the genetic background from selection at other loci (Carlborg *et al.* 2006; Le Rouzic *et al.* 2007; Le Rouzic and Carlborg 2008). However, for such an effect to explain the observations, the allele frequency change at the capacitating locus must also have been very rapid, and there is no evidence of such an event. Similar arguments can be applied to other selection modifying factors as well; there is no indication of any event that drastically changed the fitness landscape in the recent past.

The alternative is the occurrence of a novel, strongly selected mutation. The likelihood of such a mutation is difficult to assess because it depends on the target size for beneficial mutations, the mutation rate and distribution of fitness effects, neither of which is known or can be estimated from the available data because there are, to our knowledge, no mutation accumulation studies performed in comparable populations. However, given the pattern of heterozygosity changes observed, a new beneficial mutation in the HWS line seems the most parsimonious explanation. Regardless of the cause, the pattern of loss of heterozygosity implies that the selected element reached fixation at some point between generations 45 and 50.

### Divergence between selected and relaxed lines

Whole-genome comparisons between the relaxed lines and their corresponding selected lines (Figure 4) indicate that the LWR/LWS pair displays a larger global divergence than the HWR/HWS pair. This suggests purifying selection on alleles that decrease the fitness of the birds from the population when no longer subject to artificial selection. The high level of global divergence, in combination with the absence of large changes in allele frequencies in any particular regions, implies that many regions spread across the genome are affected, albeit each of them to a moderate degree, reaffirming the conclusion from previous studies (Johansson *et al.* 2010; Jacobsson *et al.* 2005) that there is a substantial polygenic contribution to body weight and also indicating that many growth-affecting alleles have detrimental side effects.

### Formation of haplotype blocks

Of particular interest in this study are the LD-blocks that are unique to either the LWS or the HWS, because they are most likely to reflect important events during the formation of the lineages. We observe that such blocks exist in regions inside previously identified QTL (Figure 5) (Wahlberg *et al.* 2009; Pettersson *et al.* 2011), an observation that makes these blocks interesting for further analyses to find the causative mutation underlying the signal in that genomic segment underlying the QTL peak.

### Implications for studies on the genetic architecture of body size

The results reported in this study are well in line with the general consensus regarding the genetic architecture of body size in that a large number of genes contribute intermediate to small individual effects and that a smaller number of loci display larger effects (Hu *et al.* 2007; Lango Allen *et al.* 2010; Visscher *et al.* 2010). Support for such conclusions can be found, *e.g.*, in the results compiled in the AnimalQTLdb (Hu *et al.* 2007), which reveal that most studied traits in domestic farm animals appear to be influenced by a few loci with large effects and many more loci with smaller effects. Similarly, recent reports support a highly polygenic architecture of human height (Lango Allen *et al.* 2010; Visscher *et al.* 2010). In contrast, a number of recent studies of the genetics underlying stature in dogs (Sutter *et al.* 2007; Boyko *et al.* 2010) and horses (Makvandi-Nejad *et al.* 2012) argue that only one or a few major genes regulate the large variation in stature found in these species. This is highly surprising given the polygenic nature of similar traits across the large number of studies in other species. It is known that the importance of individual loci detected in genetic mapping studies is often overestimated, and thus it appears premature to draw general conclusions about the genetic architecture of stature in these species based on the results of the studies performed to date. Naturally, differences can also arise because of the differences in the selection regimes to which the studied organisms have been subjected. However, the extent of that effect is currently not known. Future and more powerful studies designed to also allow evaluation of the contribution of loci with smaller effects are, in our opinion, needed before certain conclusions can be drawn about the general genetic architecture or how it is influenced by different selection schemes.

## Conclusion

Understanding the genome dynamics of beneficial mutations is an important challenge in agricultural and evolutionary genetics. We have continued to dissect the footprint of genomic change throughout 53 generations of selection in the Virginia chicken lines and have shown that it is likely that the continued selection response in the HWS line is attributable to standing genetic variation present in the base population and also stems from a novel mutation with large effect. The observed pattern of changes is consistent with a mutation subject to very strong selection appearing at a point somewhere between generation 40 and 45, an interesting example of how novel mutations can contribute to selection response during long-term selection.

The evidence is weak for purging selection at individual loci in the lines undergoing relaxed selection, but the overall genome dynamics indicates that there is a general trend, particularly in the relaxed line derived from the low-weight line. The results support the view that body size in animals is a trait determined by a few genes with medium to strong effect acting together with many loci with small effect.

## Supplementary Material

Supporting Information
