# Supplementary material for: Dynamics of Adaptive Alleles in Divergently Selected Body Weight Lines of Chickens
Source: G3 (Bethesda). 2013 Oct 29;3(12):2305–12. doi: 10.1534/g3.113.008375 (PMC3852392; doi:10.1534/g3.113.008375)
Supplement: Supporting Information [file supp_3_12_2305__index.html]

Dynamics of Adaptive Alleles in Divergently Selected Body Weight Lines of Chickens — Supporting Information 

# Dynamics of Adaptive Alleles in Divergently Selected Body Weight Lines of Chickens

## Supporting Information for Pettersson *et al.*, 2013

**Files in this Data Supplement:**

- Supporting Information - Files S1-S5 (description of contents). (PDF, 435 KB)
- File S1 - Chromosome-by-chromosome heterozygosity profiles for chromosomes 1-10 (.zip, 3 MB)
- File S2 - Chromosome-by-chromosome heterozygosity profiles for chromosomes 11-20 (.zip, 2 MB)
- File S3 - Chromosome-by-chromosome heterozygosity profiles for chromosomes 21-32 (.zip, 2 MB)
- File S4 - R scripts (.zip, 11 KB)
- File S5 - Genotype data and metadata (.zip, 4 MB)
