# Supplementary material for: Dynamics of Adaptive Alleles in Divergently Selected Body Weight Lines of Chickens
Source: G3 (Bethesda). 2013 Oct 29;3(12):2305–12. doi: 10.1534/g3.113.008375 (PMC3852392; doi:10.1534/g3.113.008375)
Supplement: Supporting Information [file supp_g3.113.008375_008375SI.pdf]

G3 · Genes | Genomes | Genetics

Dynamics of adaptive alleles in divergently selected body weight lines of chickens

Mats E. Pettersson[*], Anna M Johansson[§], Paul B. Siegel[†], Örjan Carlborg[*]
[*]Dept. of Clinical Sciences, Swedish University of Agricultural Sciences, Box 7054, 750 07, Uppsala, Sweden, Uppsala, Sweden
[§]Dept. of Animal Breeding and Genetics, Swedish University of Agricultural Sciences, Box 7023, 750 07, Uppsala, Sweden
[†]Dept. of Animal and Poultry Sciences, Virginia Polytechnic Institute and State University, Blacksburg, VA 24061-0306, USA

Corresponding author: Mats E Pettersson; Dept. of Clinical Sciences, Swedish University of Agricultural Sciences; Box 7054; 750 07, Uppsala, Sweden.
Telephone: +4618672004
Email: mats.pettersson@slu.se

DOI: 10.1534/g3.113.008375

Files S1-S5

Available for download at http://www.g3journal.org/lookup/suppl/doi:10.1534/g3.113.008375/-/DC1.

Files S1-S3 Chromosome-by-chromosome heterozygosity profiles.

Each figure contains three profiles for alternative measures of heterozygosity for each chromosome: the top profile illustrates the difference between the HWS and LWS lineages at generations 40, 50 and 53. The second profile shows the difference within the HWS lineage between generation 40 and 53, where regions showing significant changes in allele-frequencies are indicated by their crossing the multiple-testing corrected global two-sided significance threshold. The bottom profile illustrates the absolute heterozygosity level for all eight populations, with shading and scaling to emphasize low heterozygosity regions. Chromosome 29 is the W chromosome, 30 is the Z chromosome, chromosome 31 is linkage group LGE22C19W28_E50C23 and chromosome 32 is linkage group LGE64. File S1 contains chromosomes 1-10, File S2 contains chromosomes 11-20, and File S3 contains chromosomes 21-32.

File S4 R scripts

File S5 Genotype data and metadata
