# Supplementary figures and images for: Dynamics of Adaptive Alleles in Divergently Selected Body Weight Lines of Chickens
Source: G3 (Bethesda). 2013 Oct 29;3(12):2305–12. doi: 10.1534/g3.113.008375 (PMC3852392; doi:10.1534/g3.113.008375)

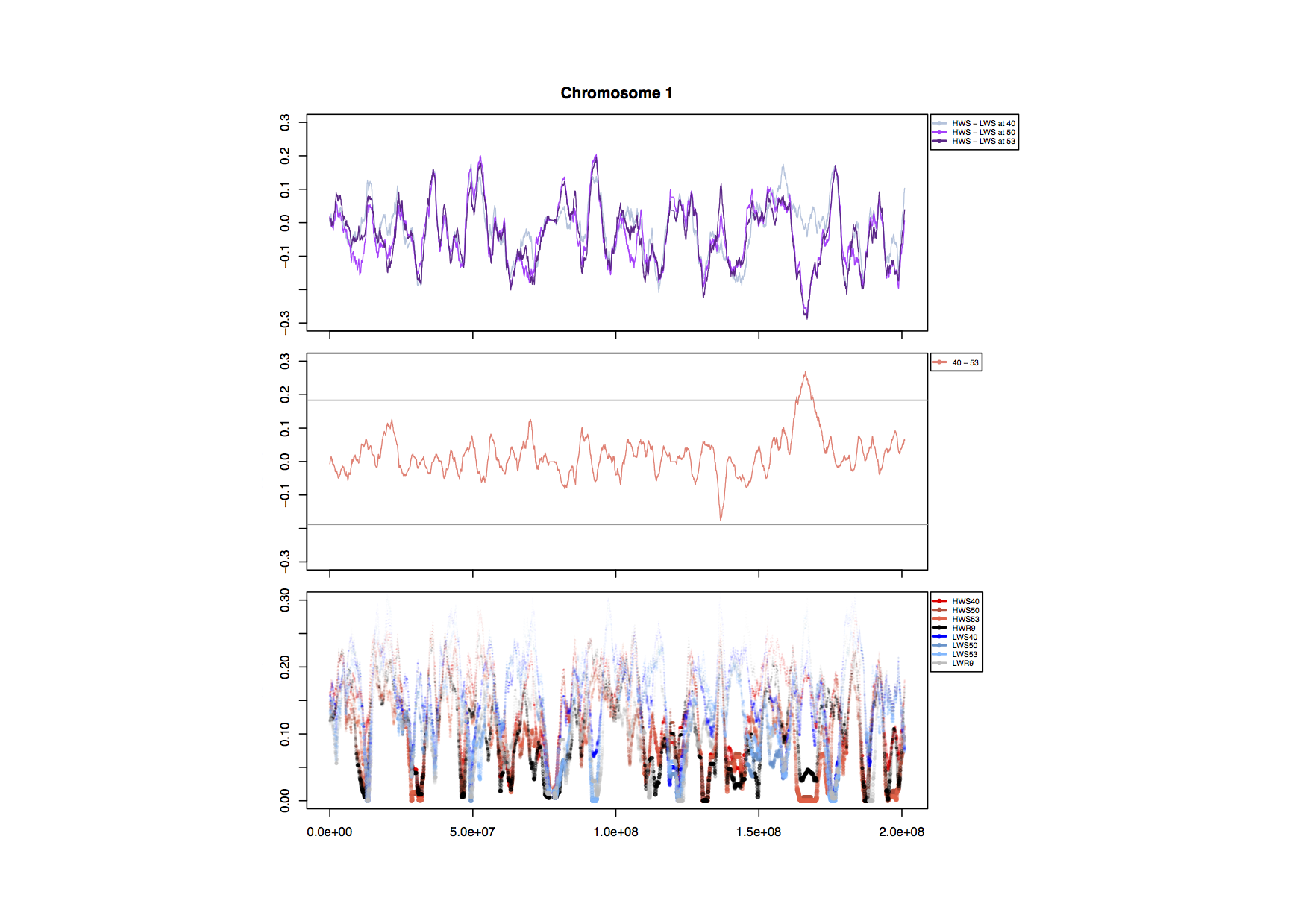

Supplement: Supporting Information [file supp_g3.113.008375_FileS1.zip › Figure_S1_chr1.tiff]

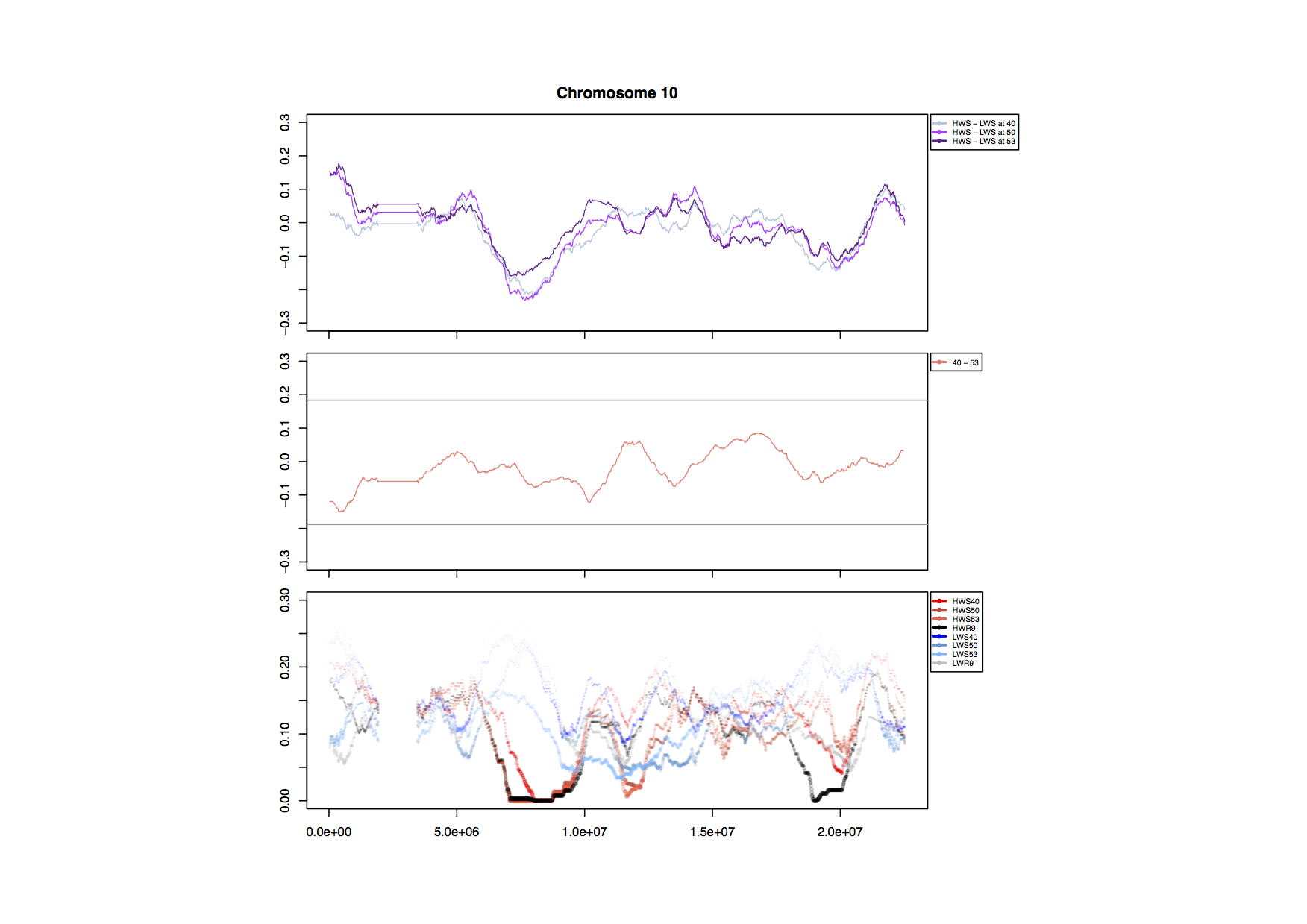

Supplement: Supporting Information [file supp_g3.113.008375_FileS1.zip › Figure_S1_chr10.tiff]

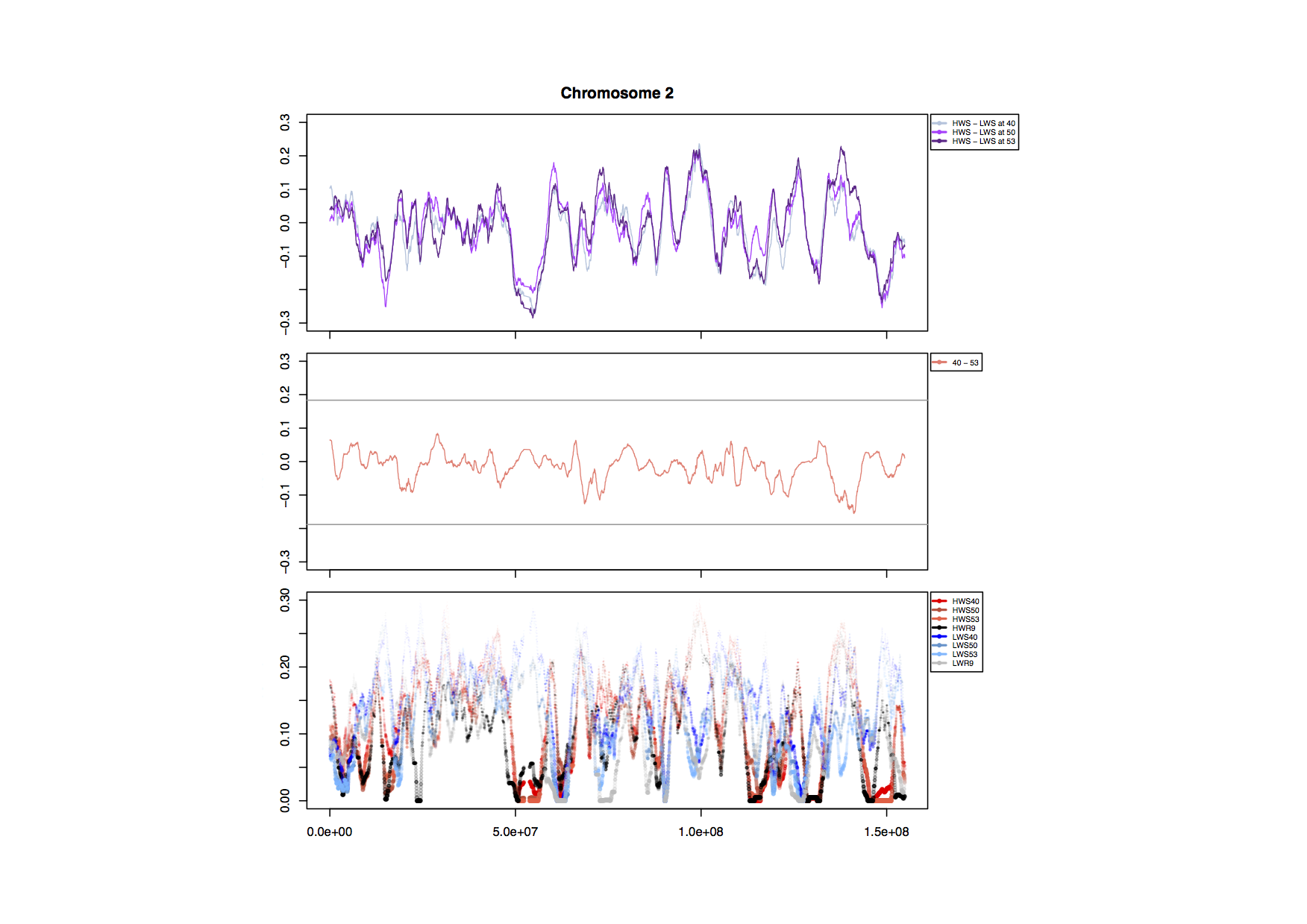

Supplement: Supporting Information [file supp_g3.113.008375_FileS1.zip › Figure_S1_chr2.tiff]

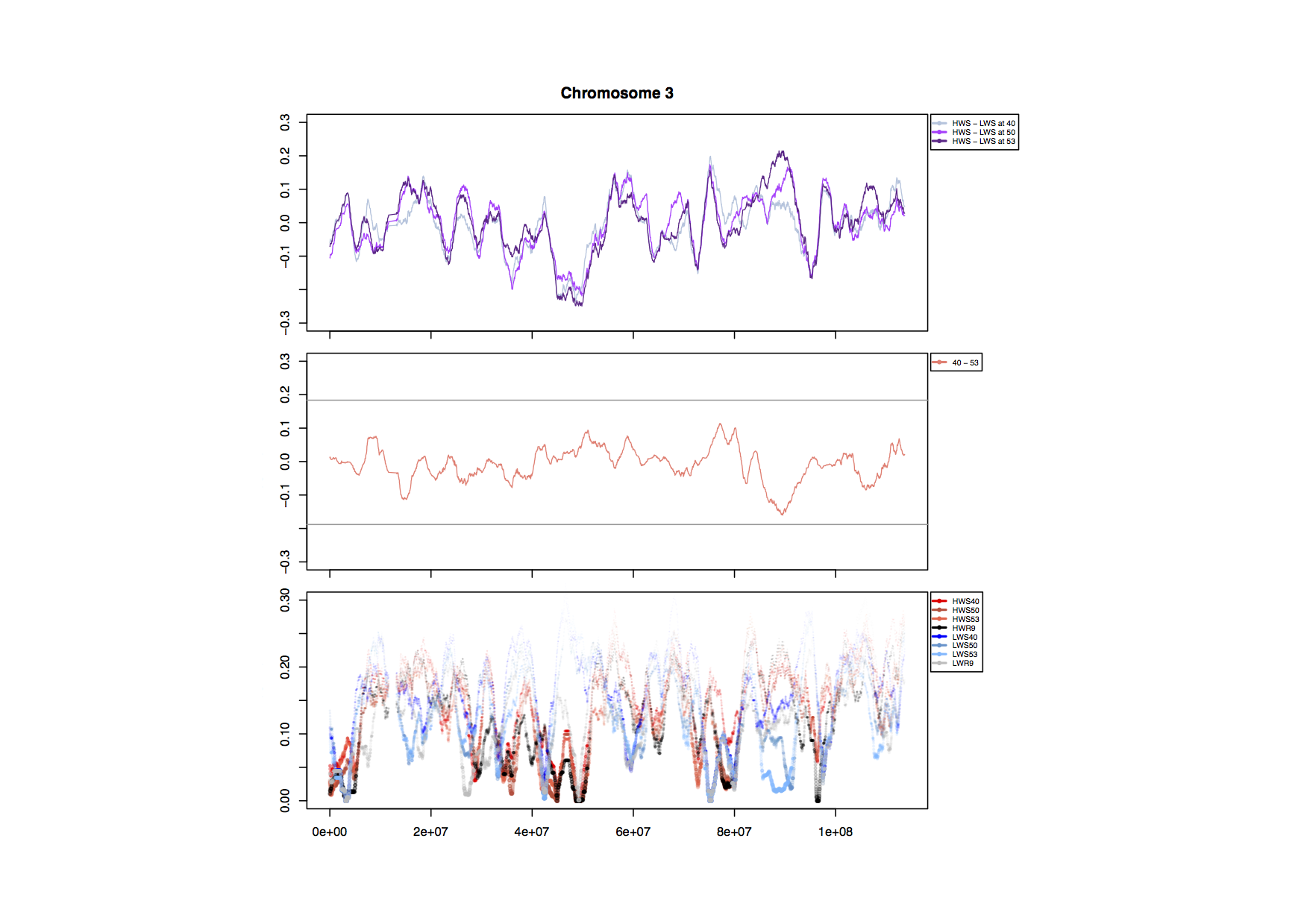

Supplement: Supporting Information [file supp_g3.113.008375_FileS1.zip › Figure_S1_chr3.tiff]

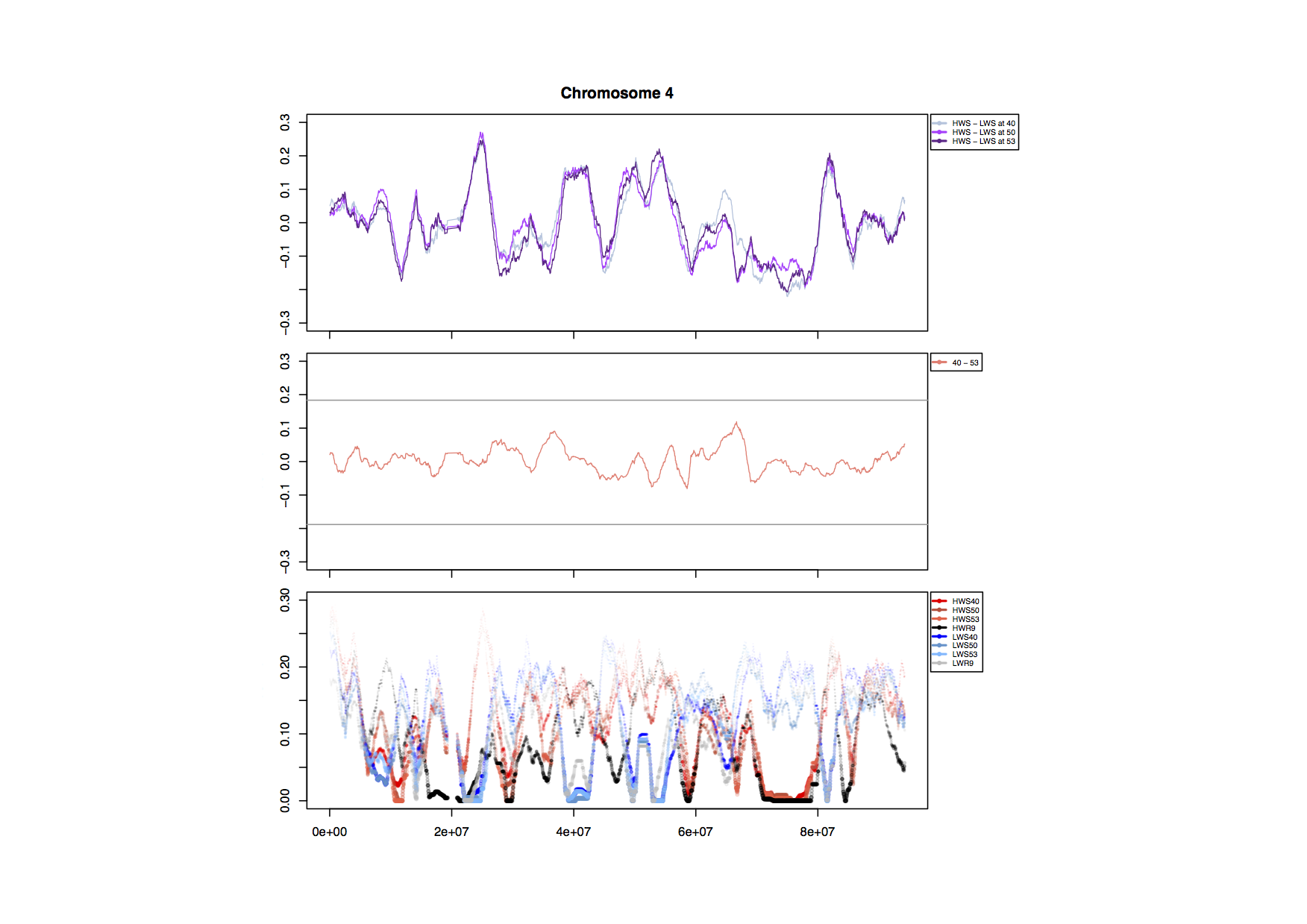

Supplement: Supporting Information [file supp_g3.113.008375_FileS1.zip › Figure_S1_chr4.tiff]

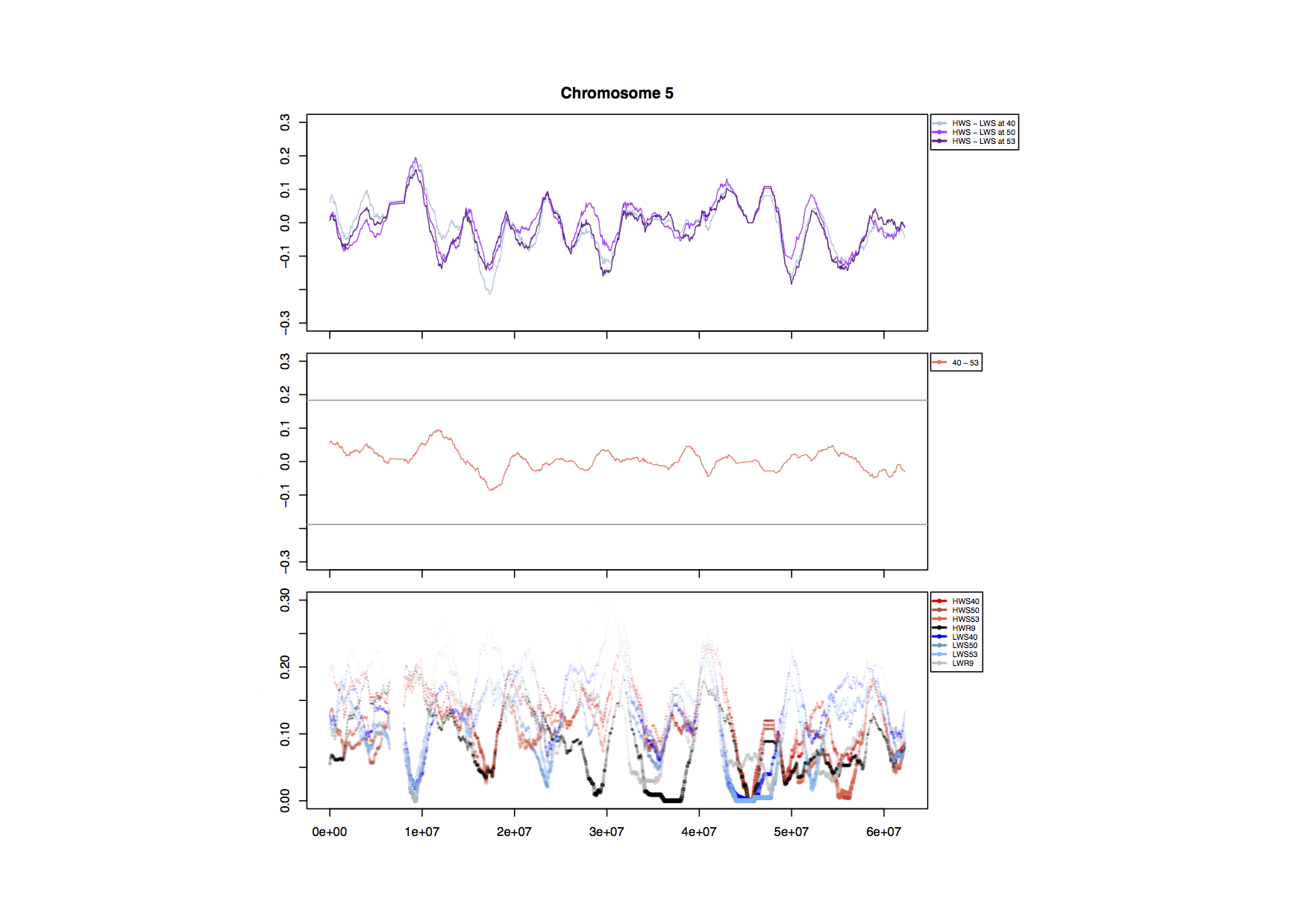

Supplement: Supporting Information [file supp_g3.113.008375_FileS1.zip › Figure_S1_chr5.tiff]

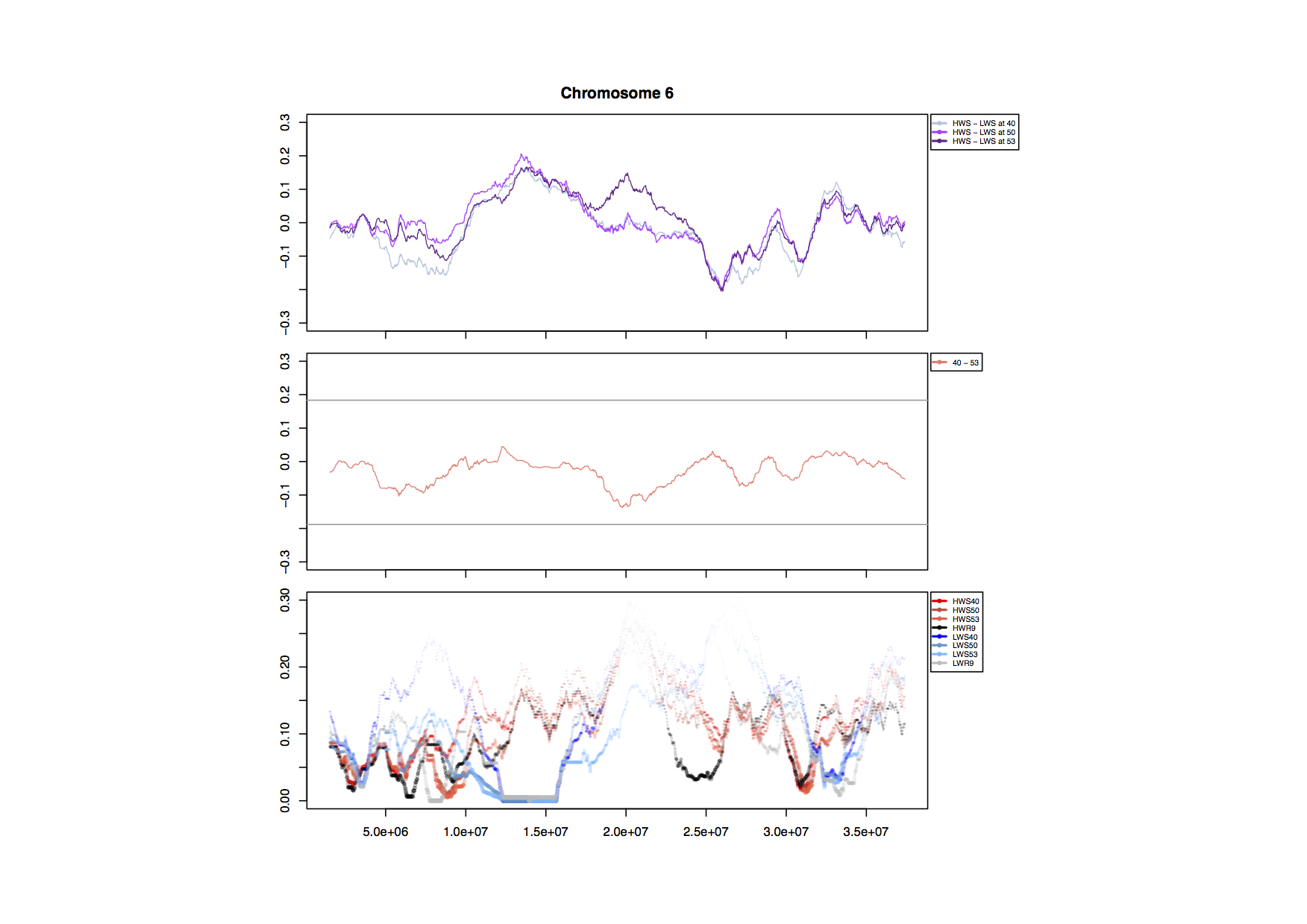

Supplement: Supporting Information [file supp_g3.113.008375_FileS1.zip › Figure_S1_chr6.tiff]

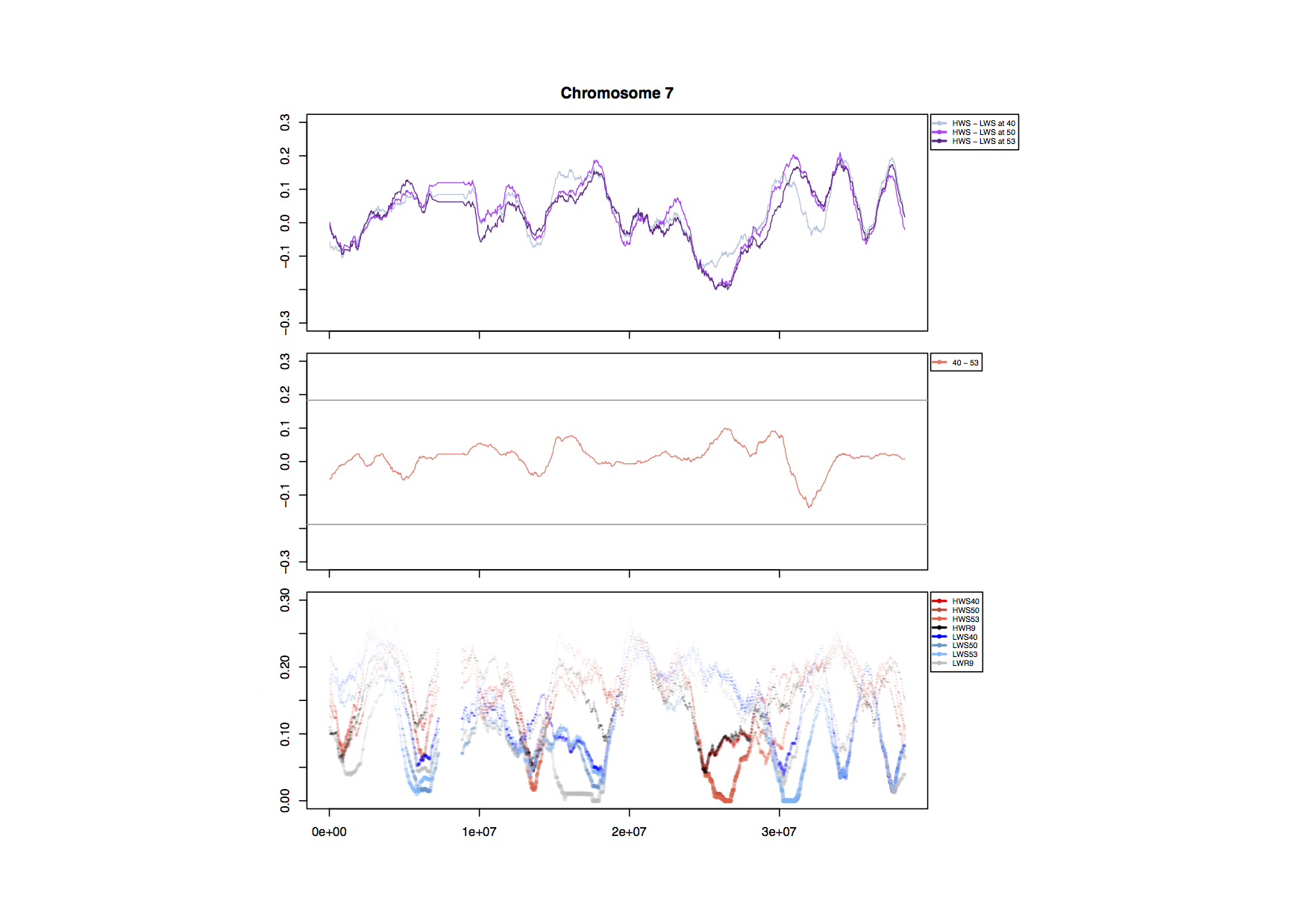

Supplement: Supporting Information [file supp_g3.113.008375_FileS1.zip › Figure_S1_chr7.tiff]

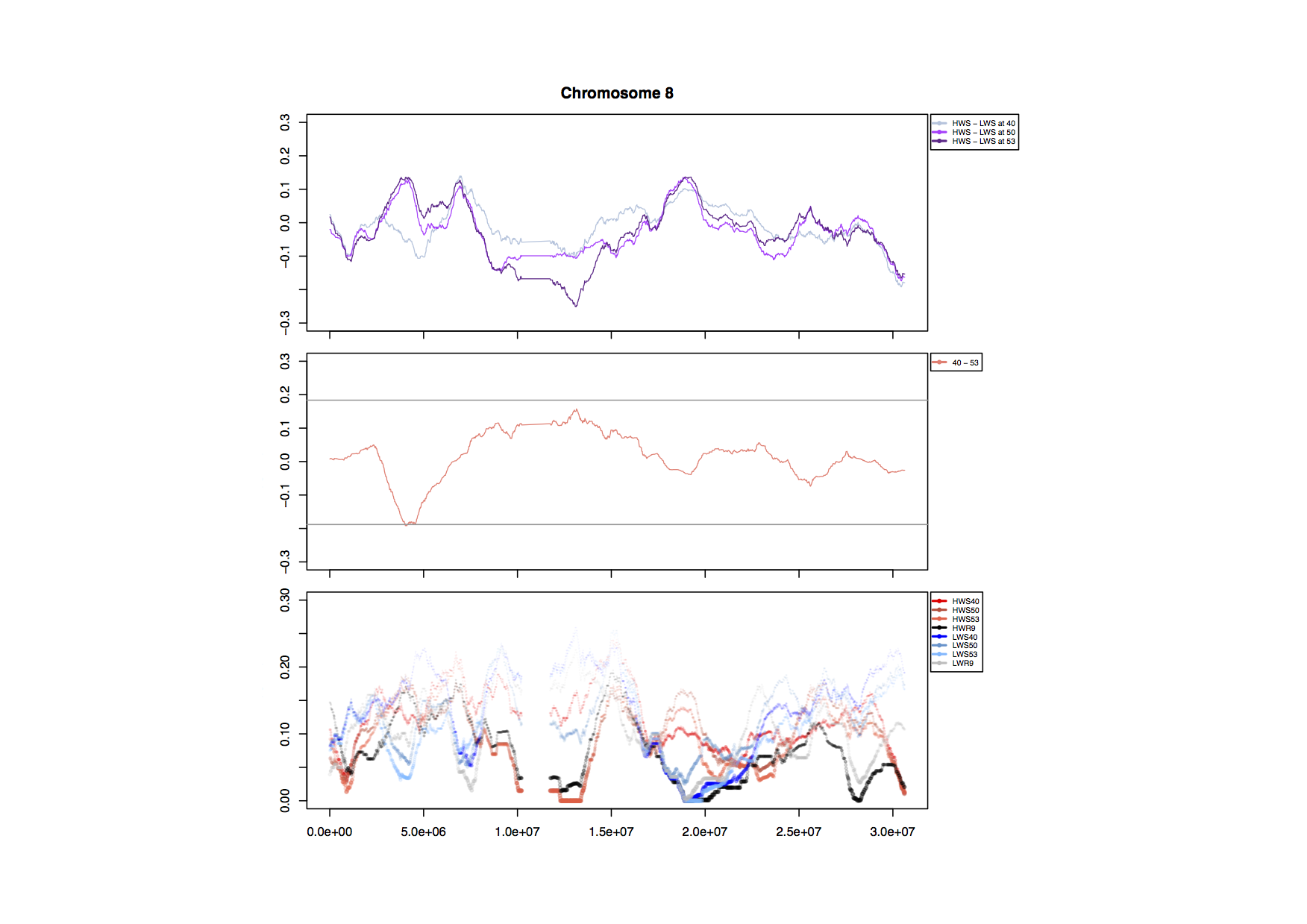

Supplement: Supporting Information [file supp_g3.113.008375_FileS1.zip › Figure_S1_chr8.tiff]

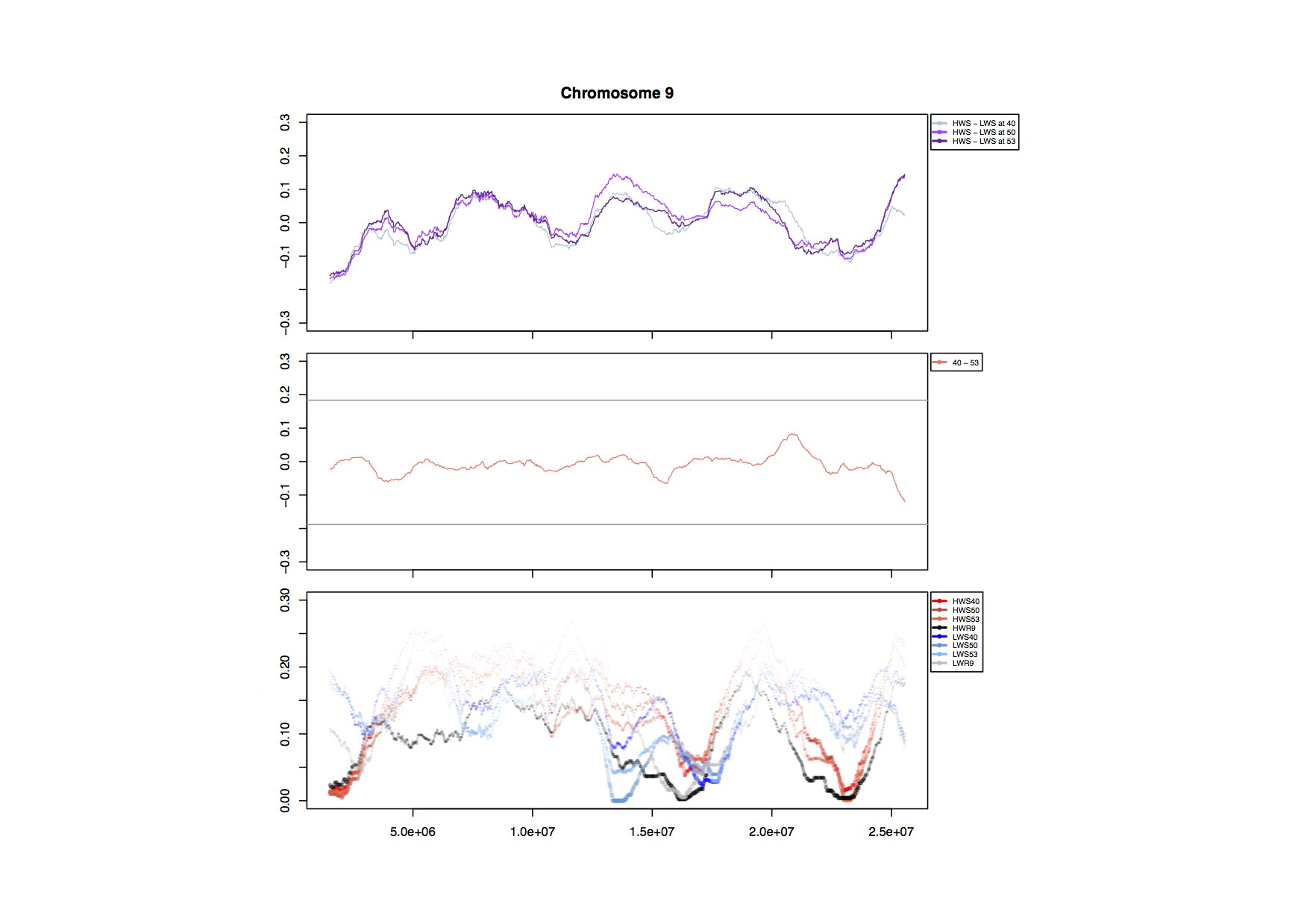

Supplement: Supporting Information [file supp_g3.113.008375_FileS1.zip › Figure_S1_chr9.tiff]

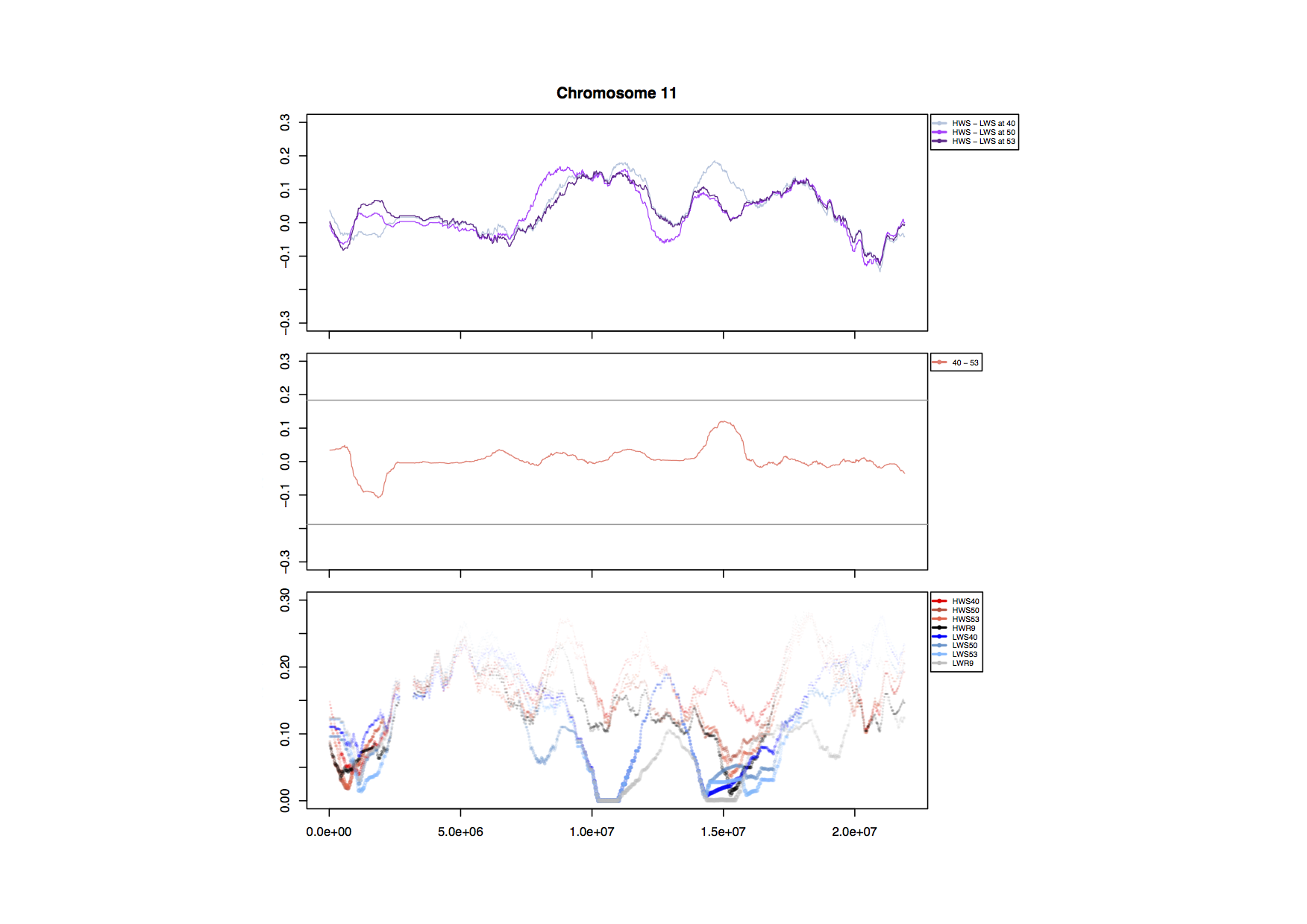

Supplement: Supporting Information [file supp_g3.113.008375_FileS2.zip › Figure_S1_chr11.tiff]

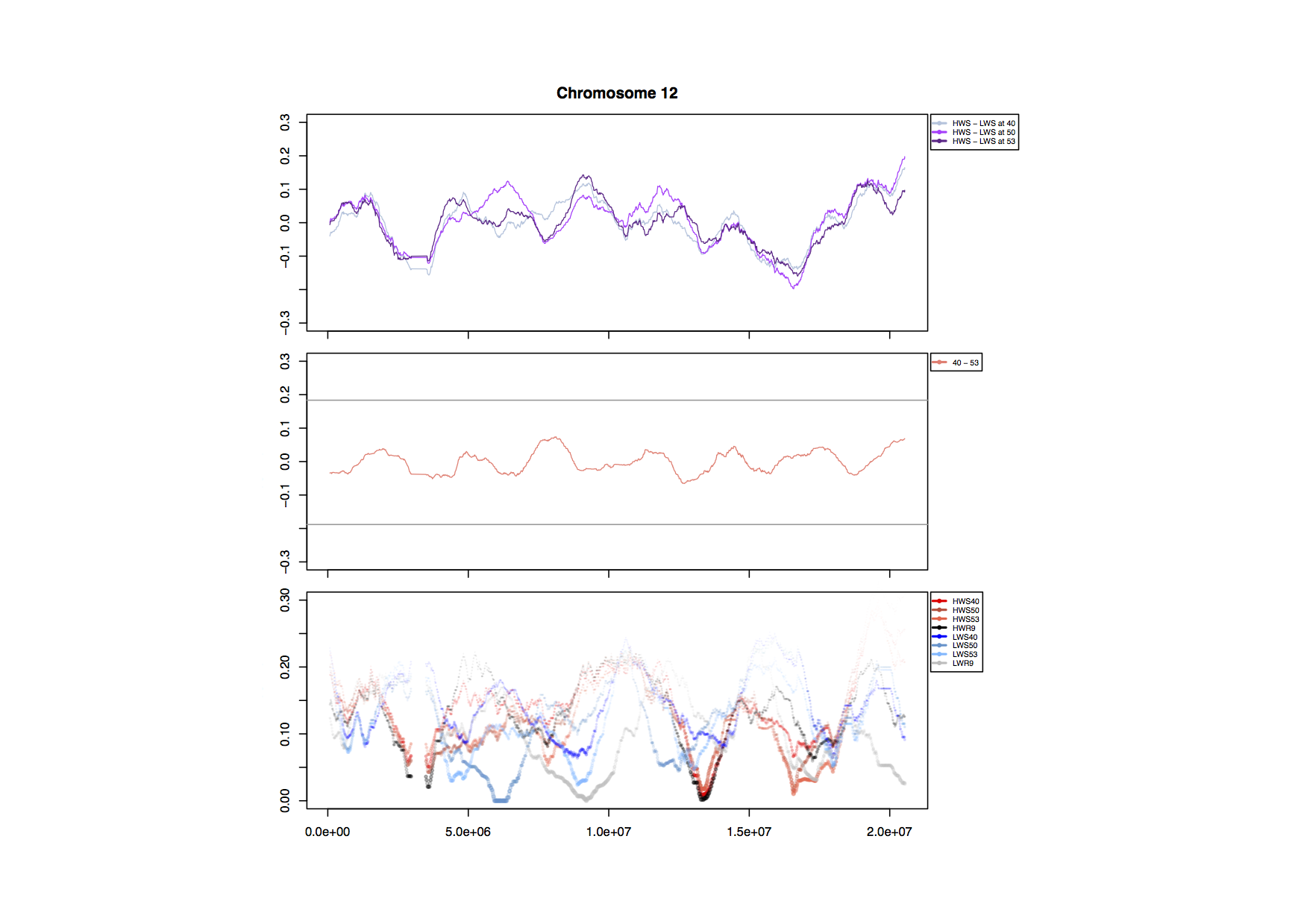

Supplement: Supporting Information [file supp_g3.113.008375_FileS2.zip › Figure_S1_chr12.tiff]

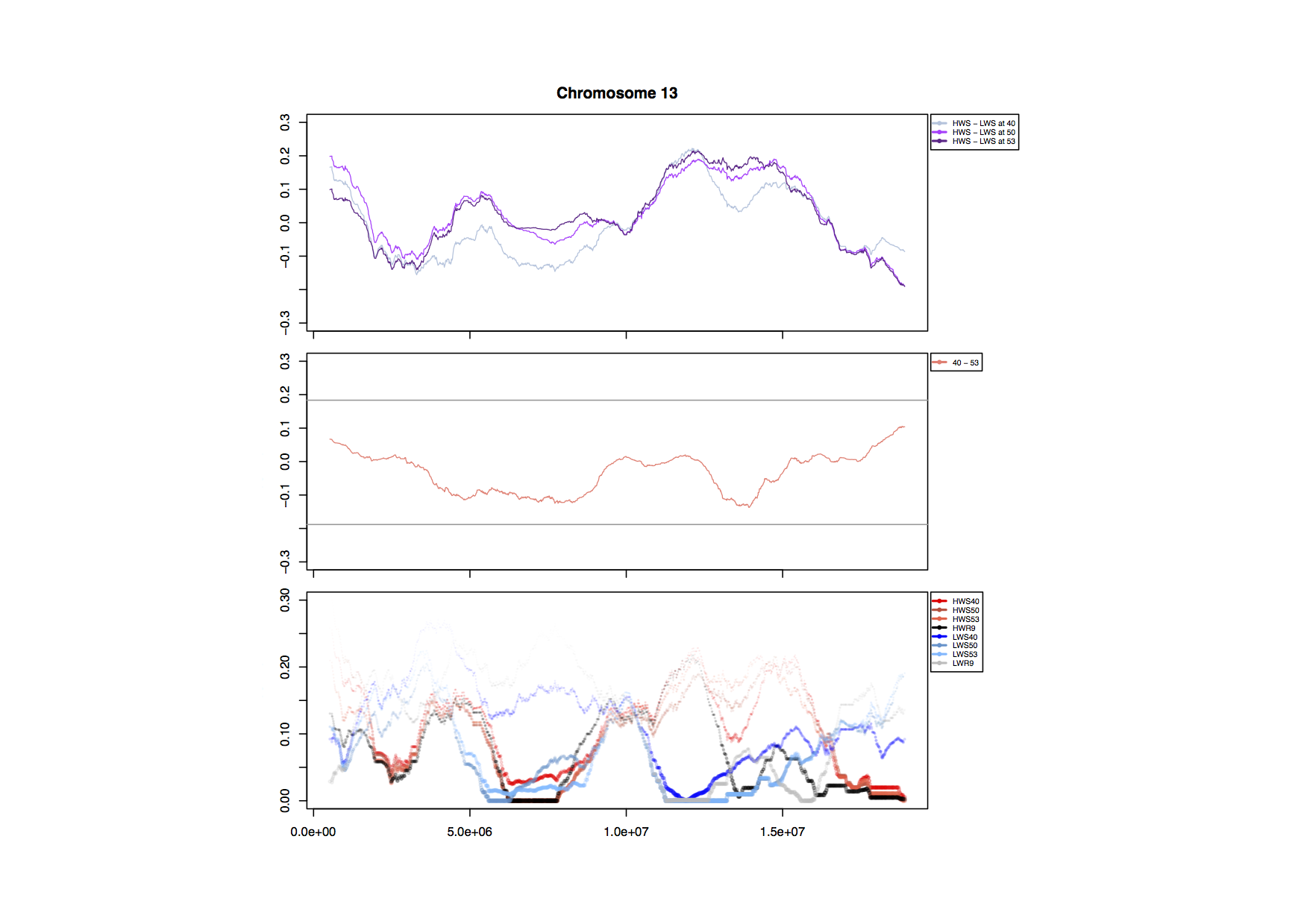

Supplement: Supporting Information [file supp_g3.113.008375_FileS2.zip › Figure_S1_chr13.tiff]

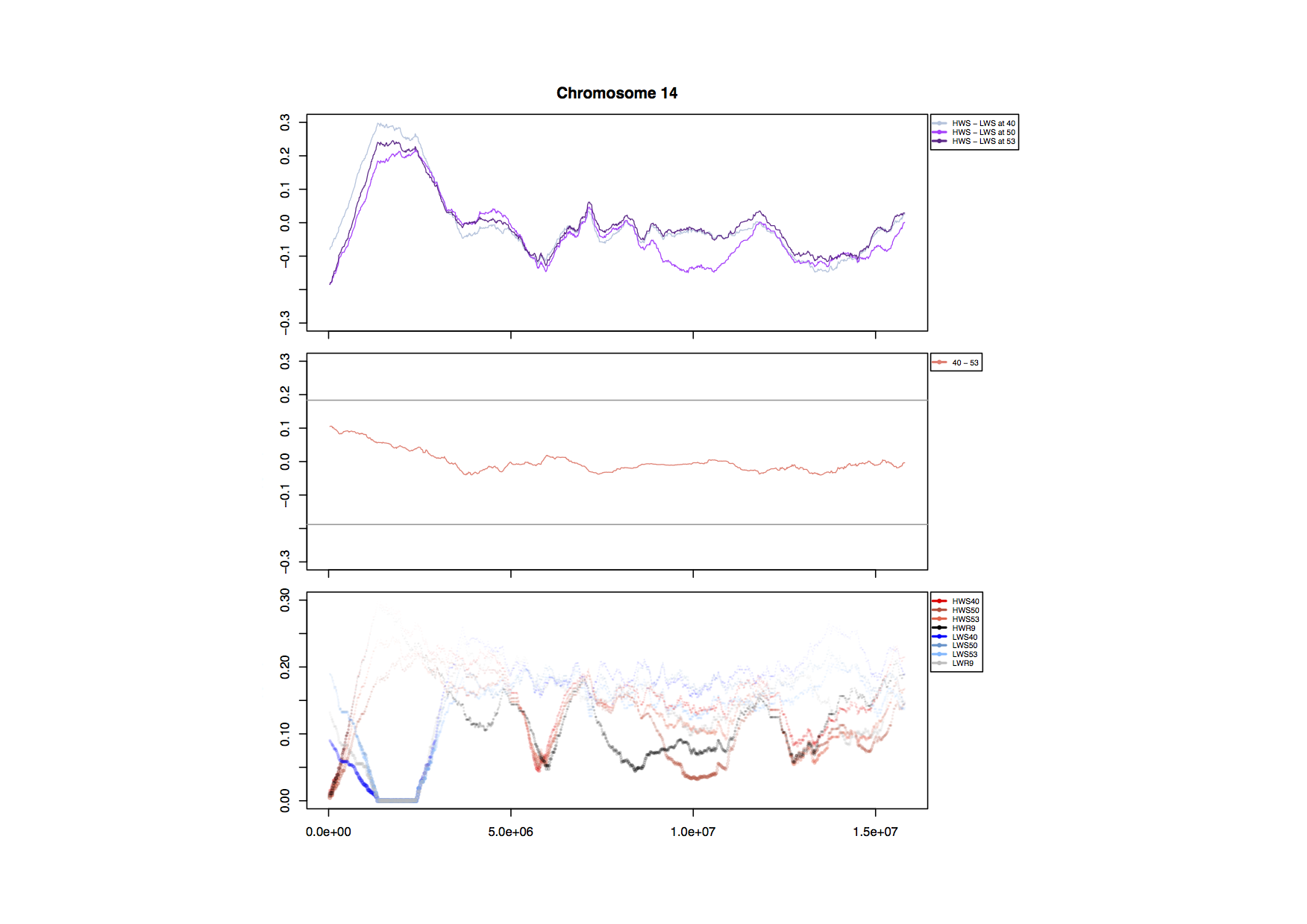

Supplement: Supporting Information [file supp_g3.113.008375_FileS2.zip › Figure_S1_chr14.tiff]

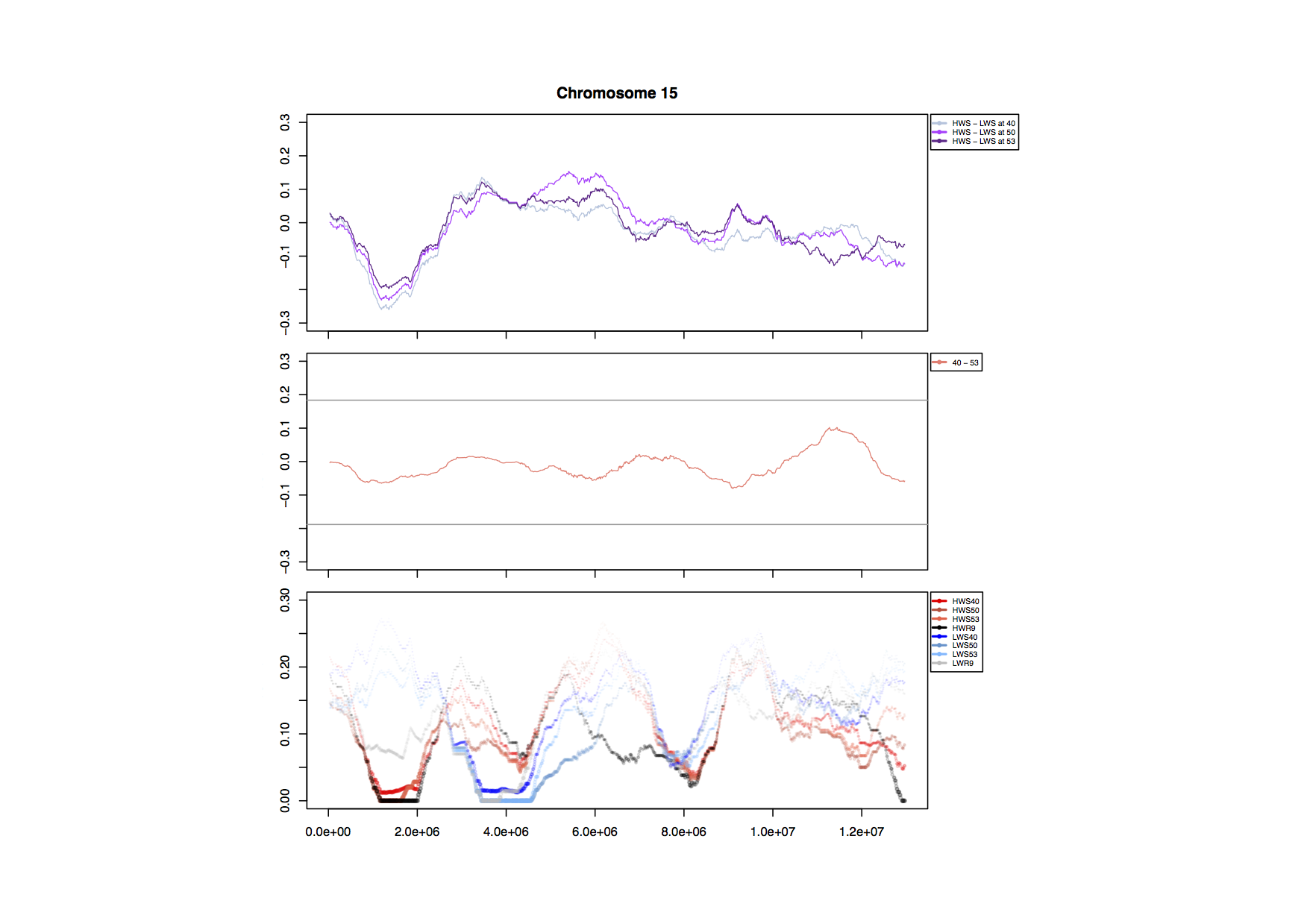

Supplement: Supporting Information [file supp_g3.113.008375_FileS2.zip › Figure_S1_chr15.tiff]

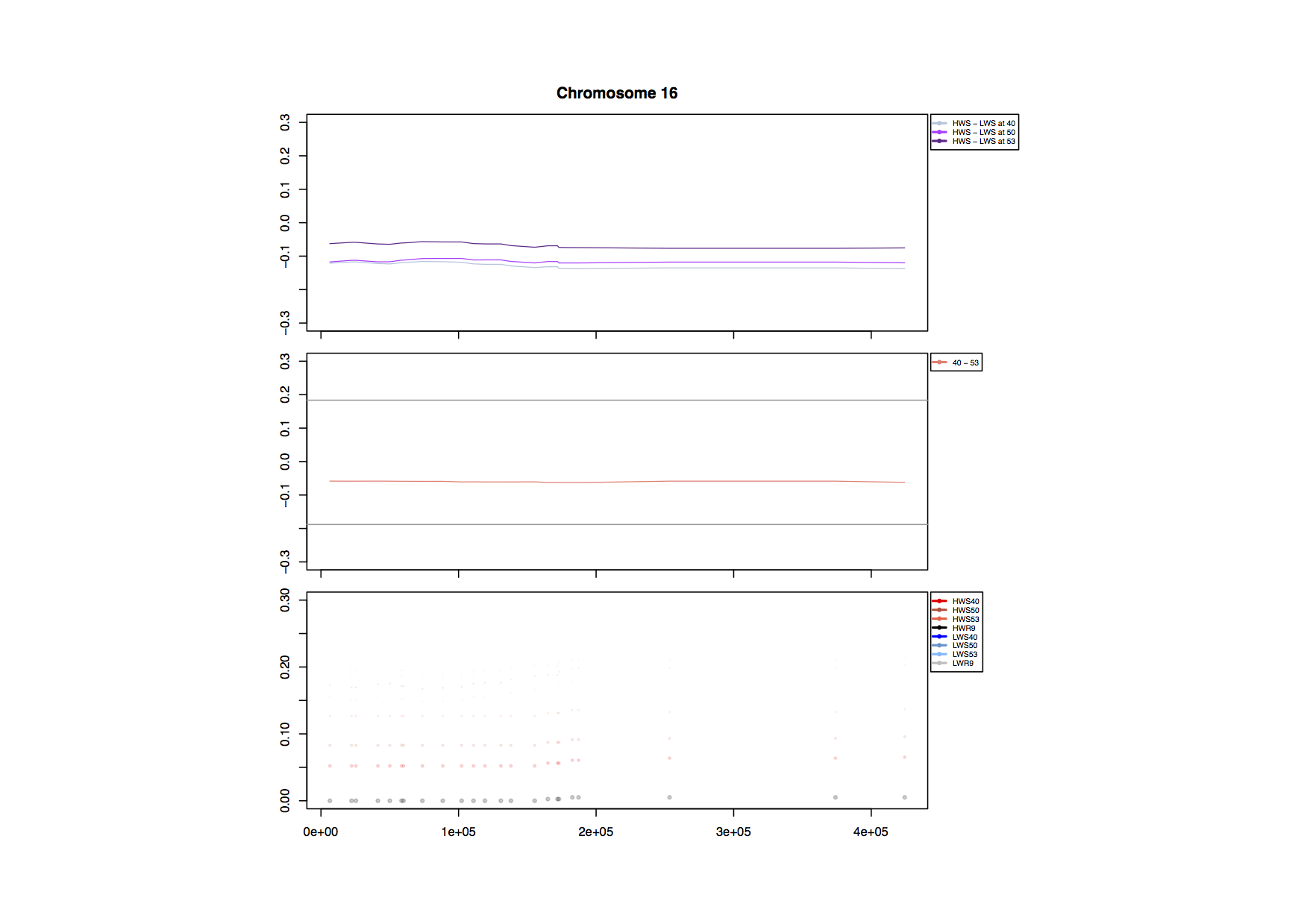

Supplement: Supporting Information [file supp_g3.113.008375_FileS2.zip › Figure_S1_chr16.tiff]

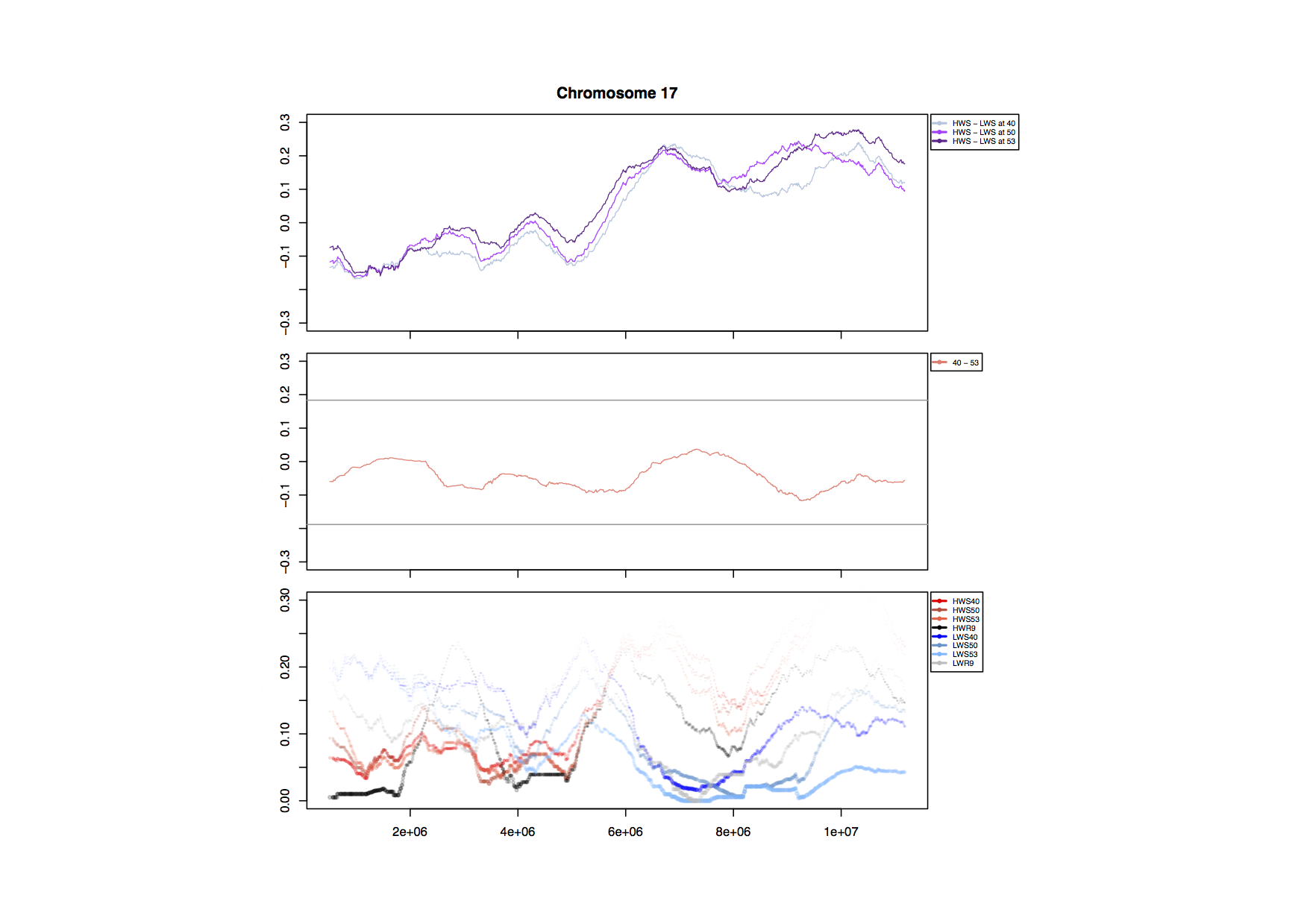

Supplement: Supporting Information [file supp_g3.113.008375_FileS2.zip › Figure_S1_chr17.tiff]

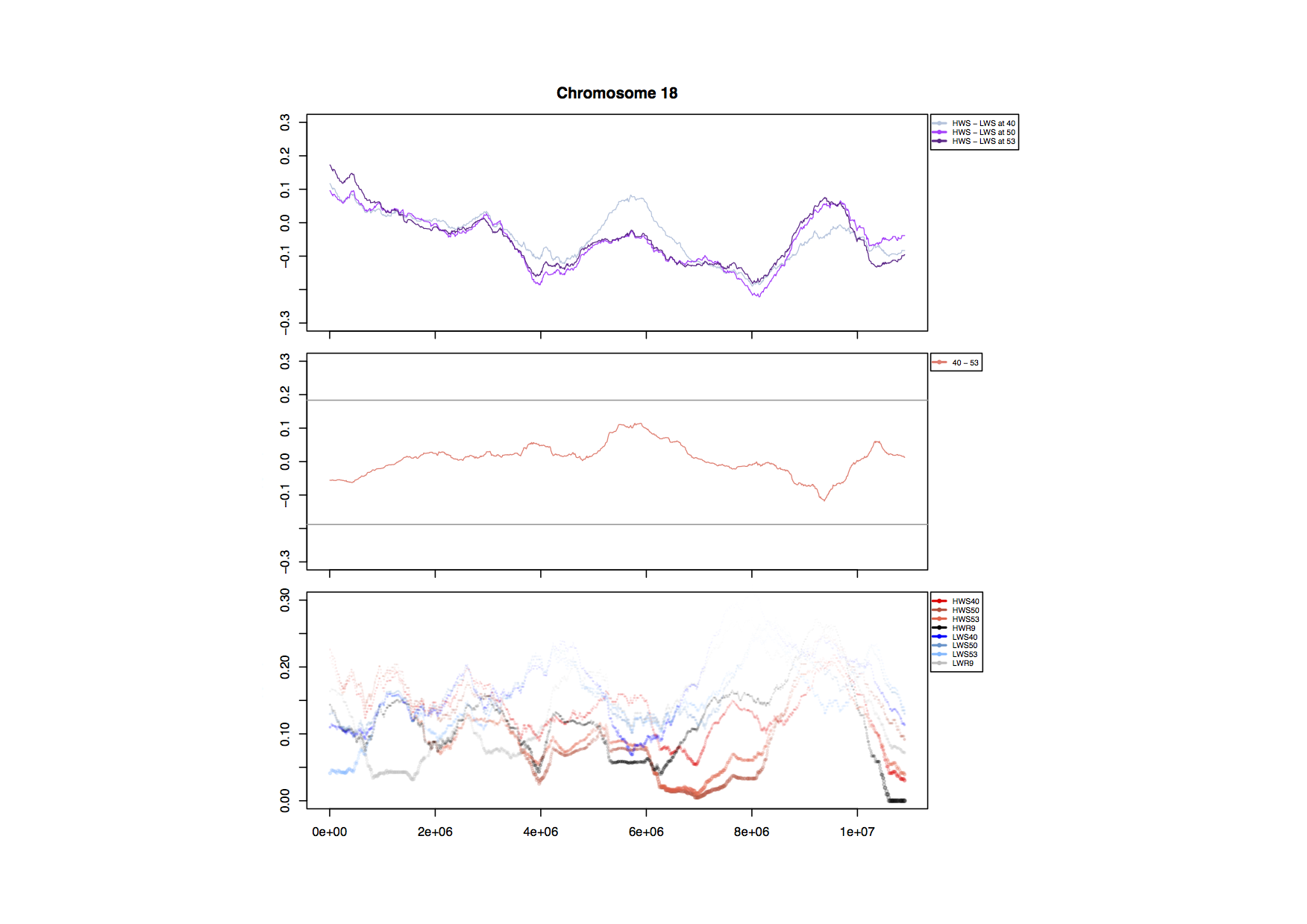

Supplement: Supporting Information [file supp_g3.113.008375_FileS2.zip › Figure_S1_chr18.tiff]

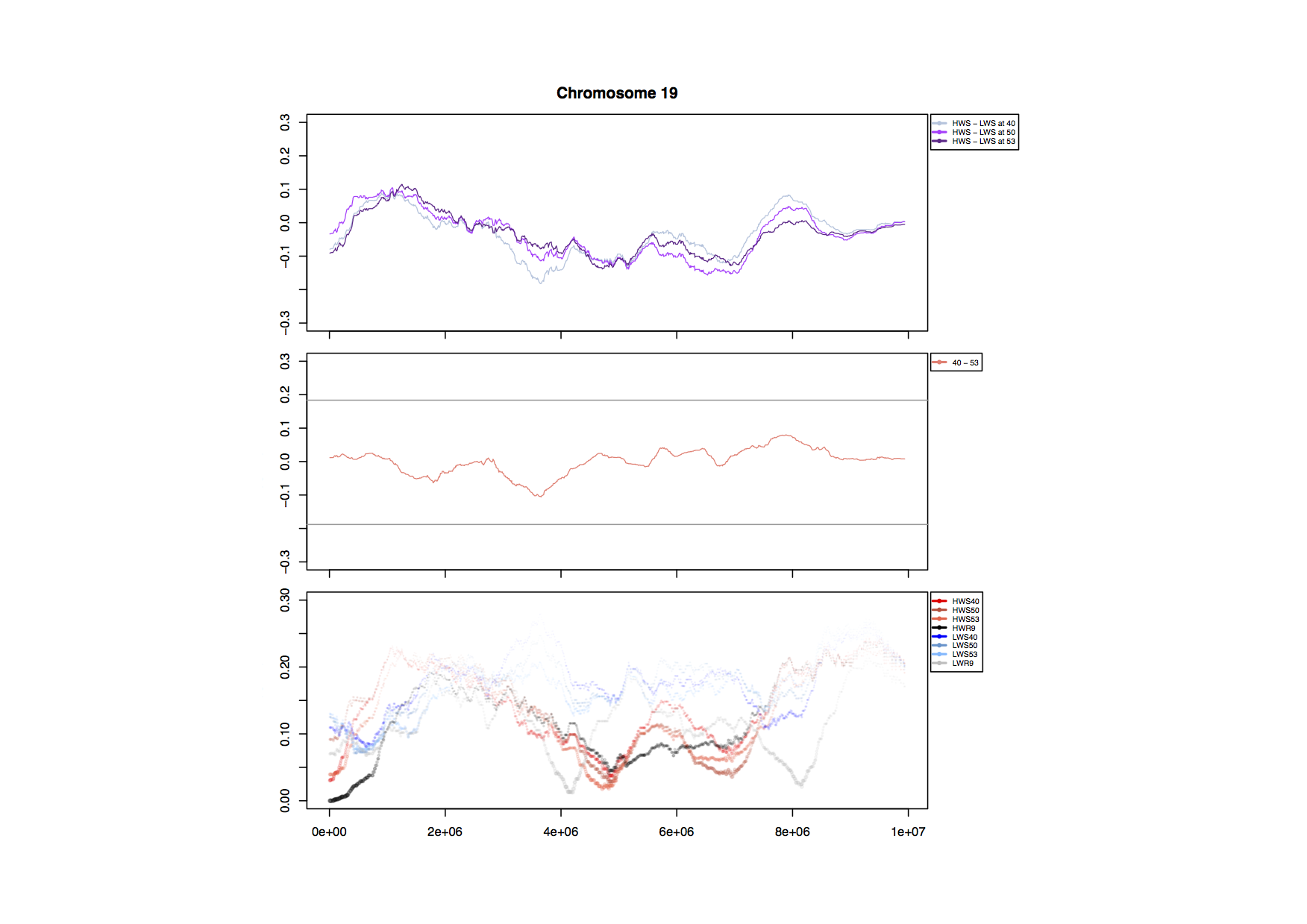

Supplement: Supporting Information [file supp_g3.113.008375_FileS2.zip › Figure_S1_chr19.tiff]

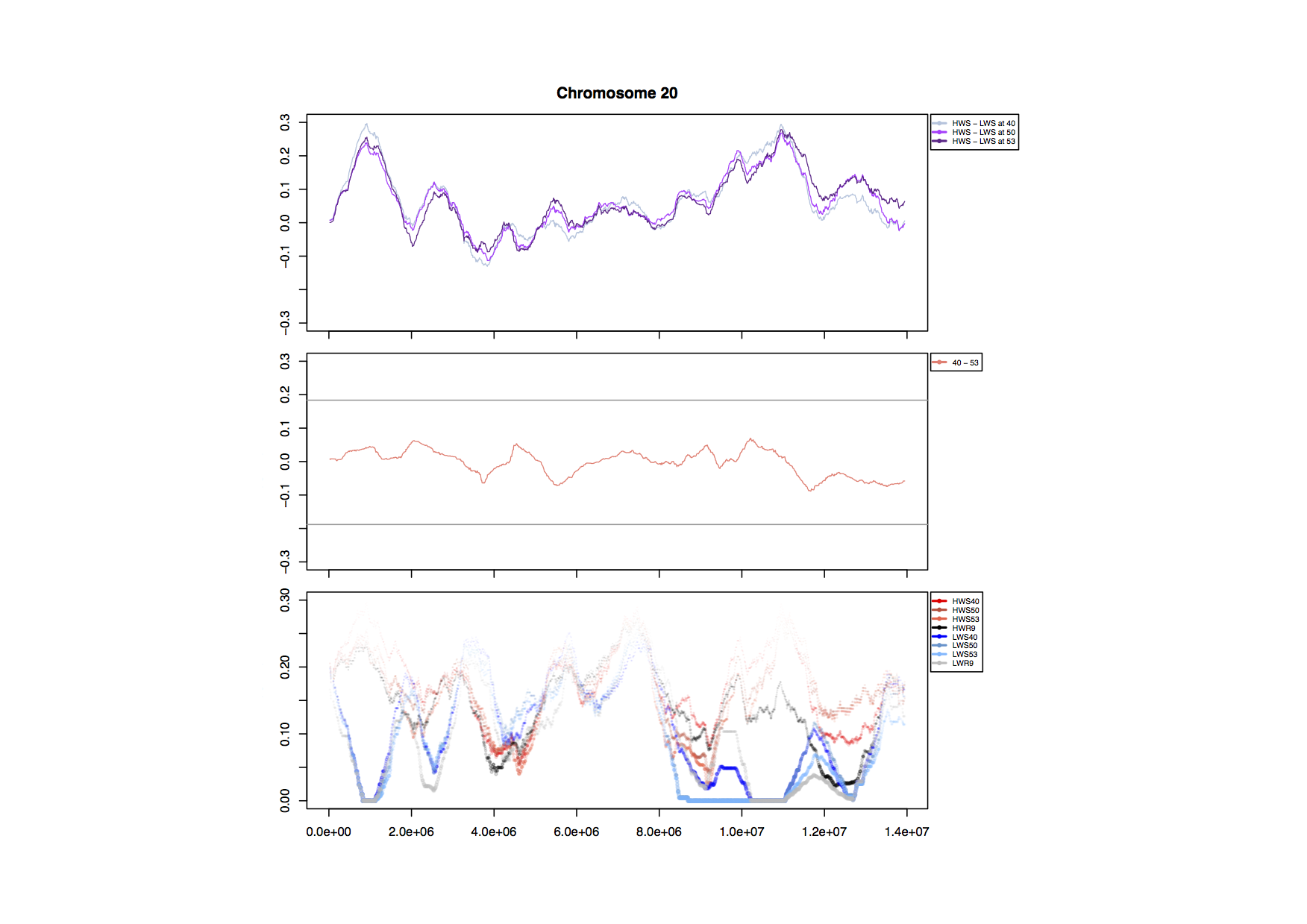

Supplement: Supporting Information [file supp_g3.113.008375_FileS2.zip › Figure_S1_chr20.tiff]

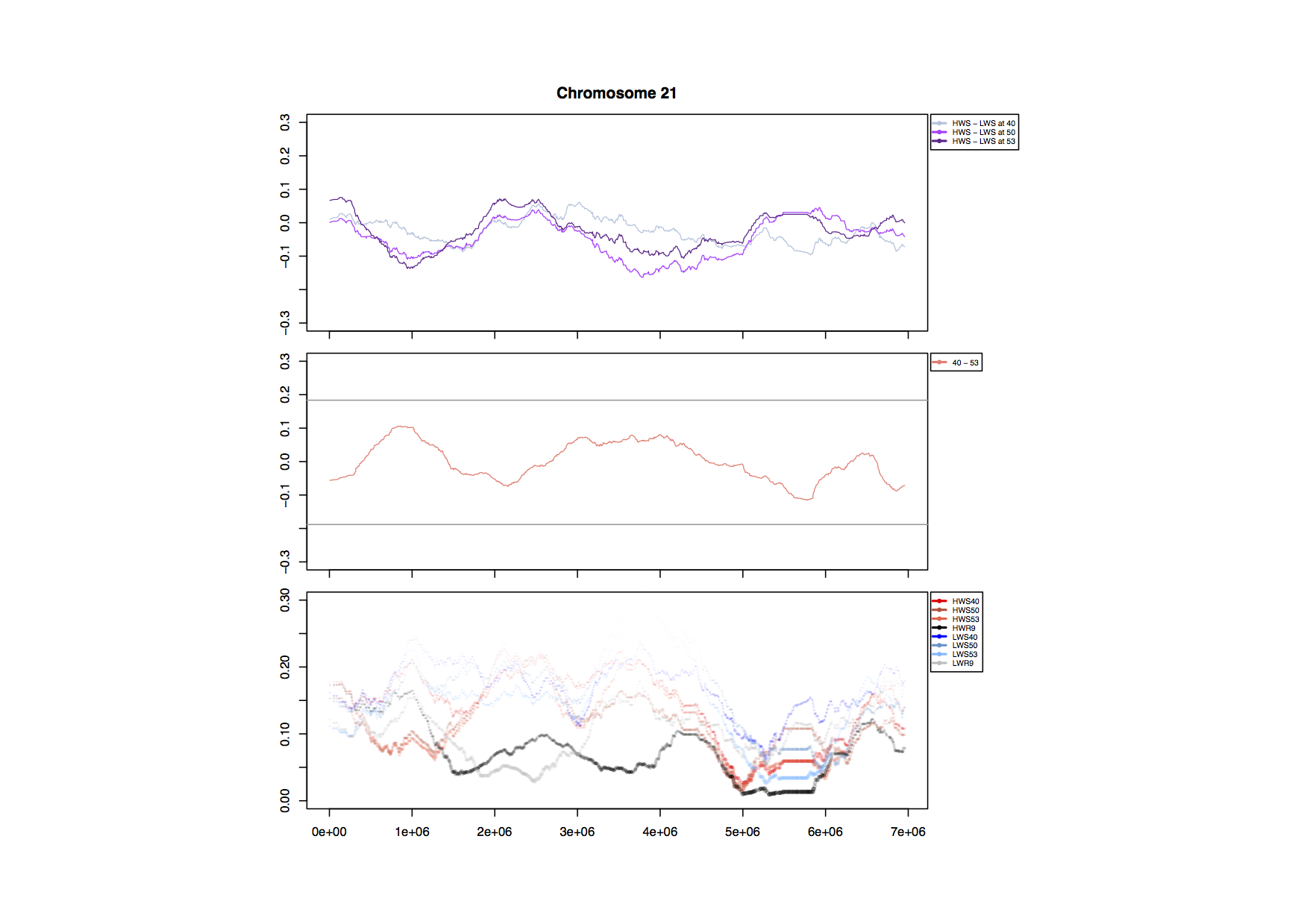

Supplement: Supporting Information [file supp_g3.113.008375_FileS3.zip › Figure_S1_chr21.tiff]

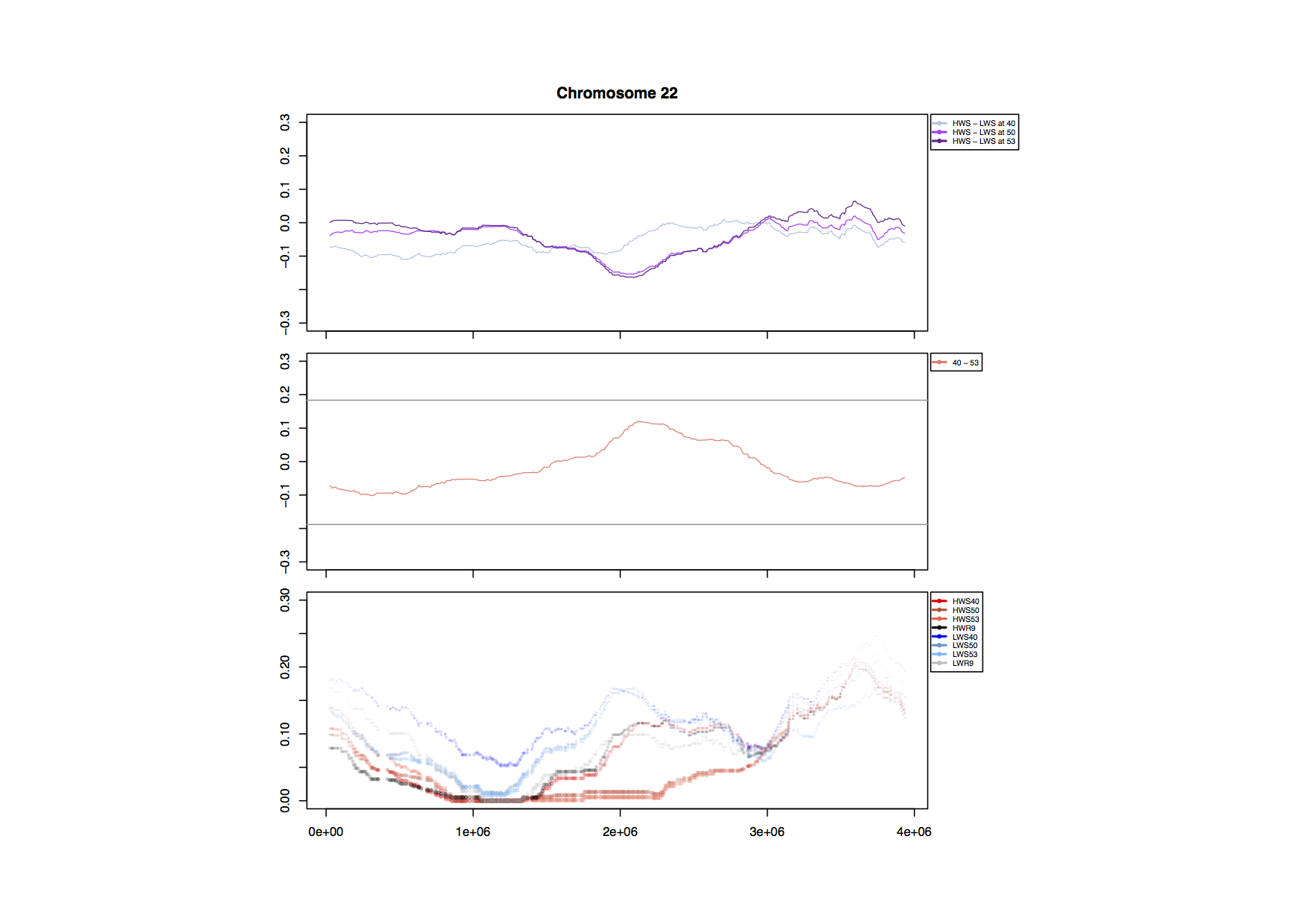

Supplement: Supporting Information [file supp_g3.113.008375_FileS3.zip › Figure_S1_chr22.tiff]

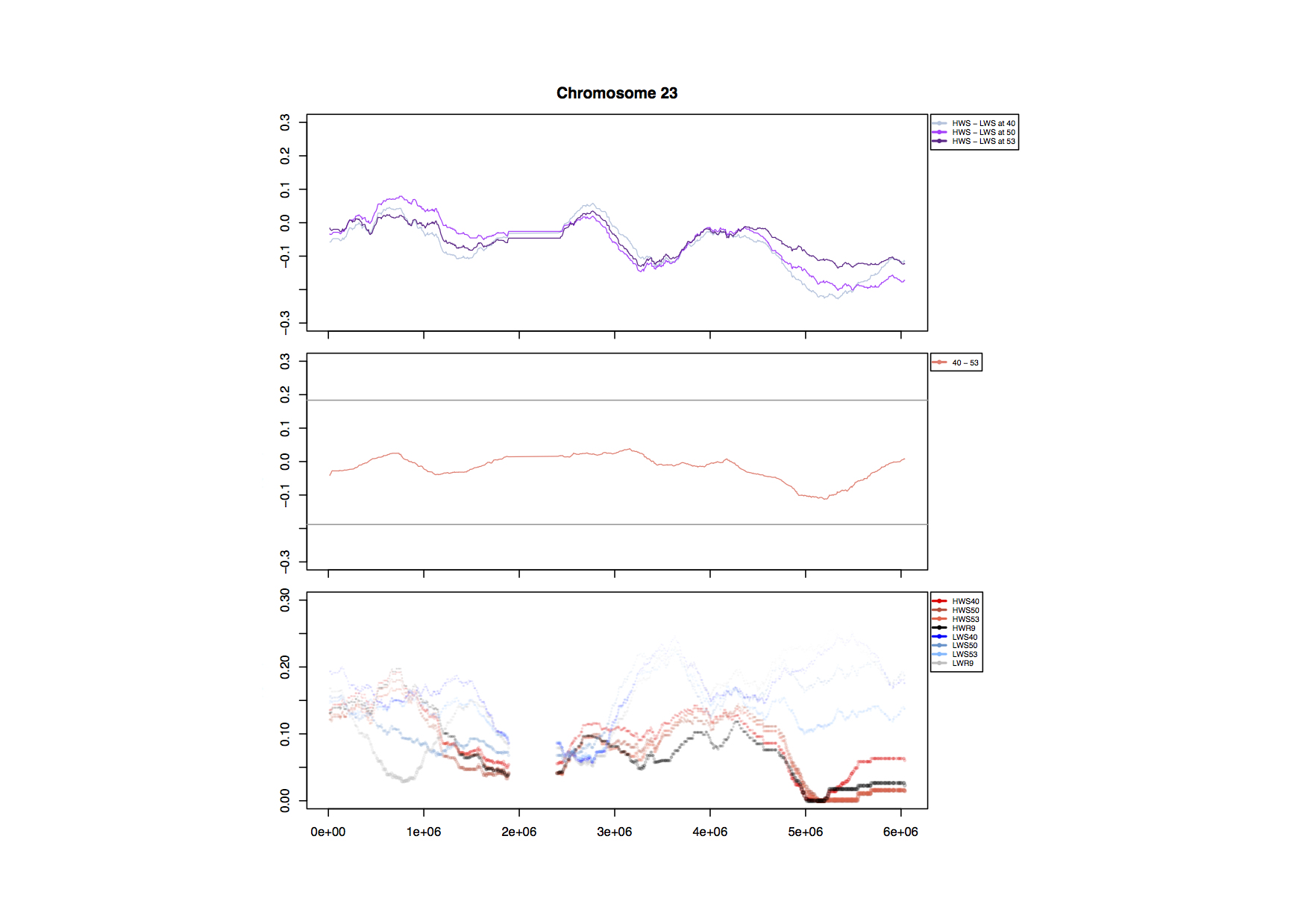

Supplement: Supporting Information [file supp_g3.113.008375_FileS3.zip › Figure_S1_chr23.tiff]

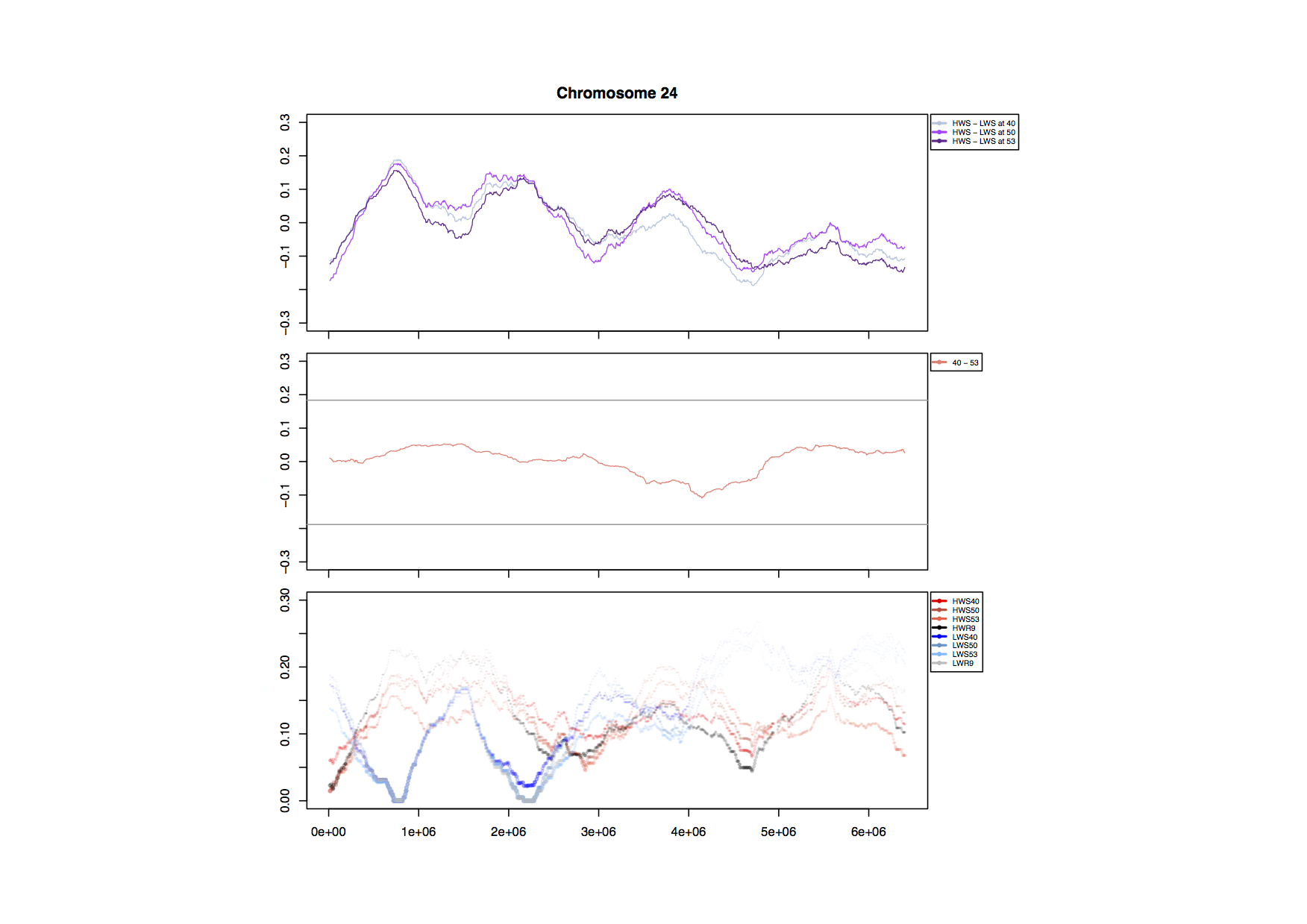

Supplement: Supporting Information [file supp_g3.113.008375_FileS3.zip › Figure_S1_chr24.tiff]

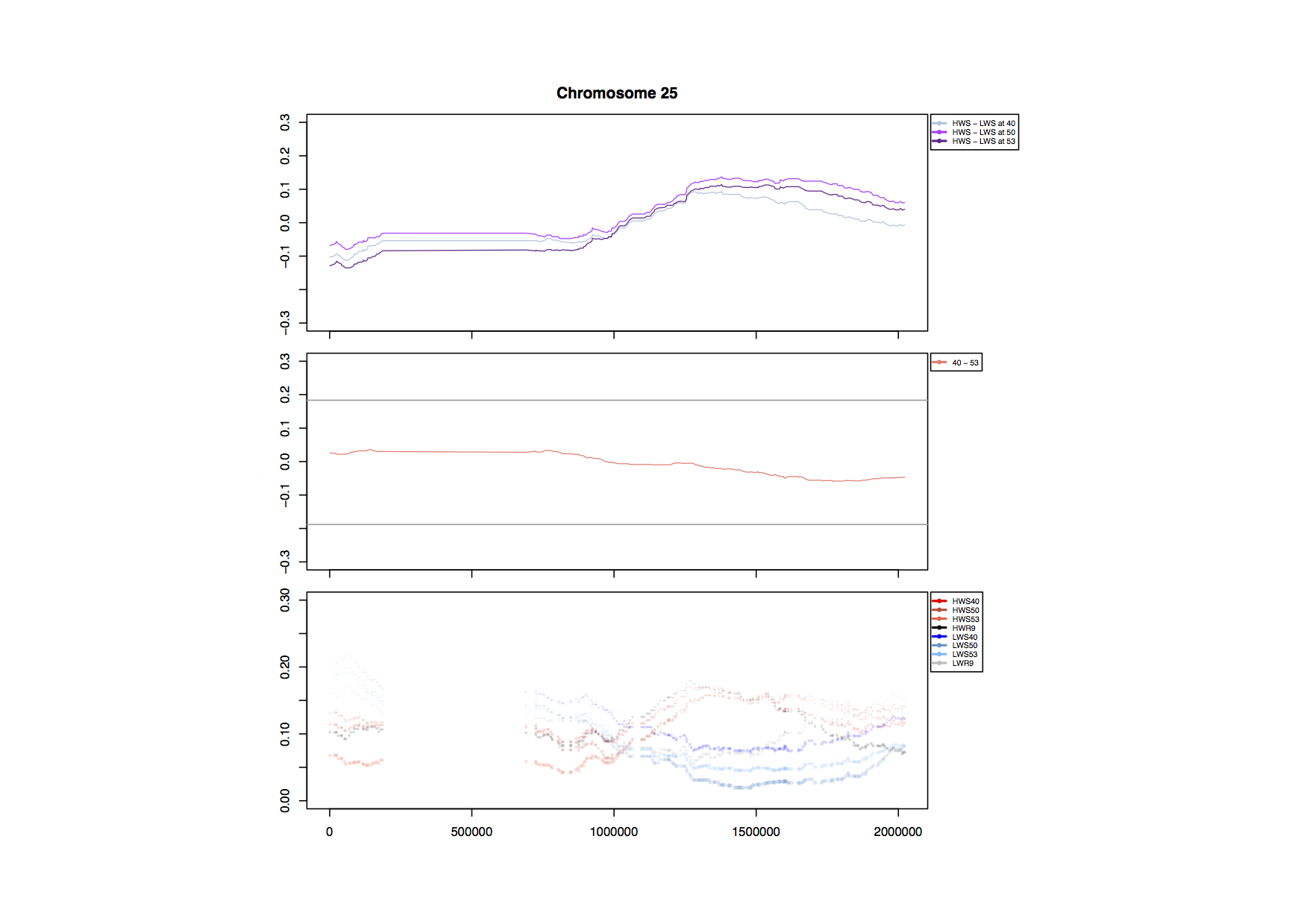

Supplement: Supporting Information [file supp_g3.113.008375_FileS3.zip › Figure_S1_chr25.tiff]

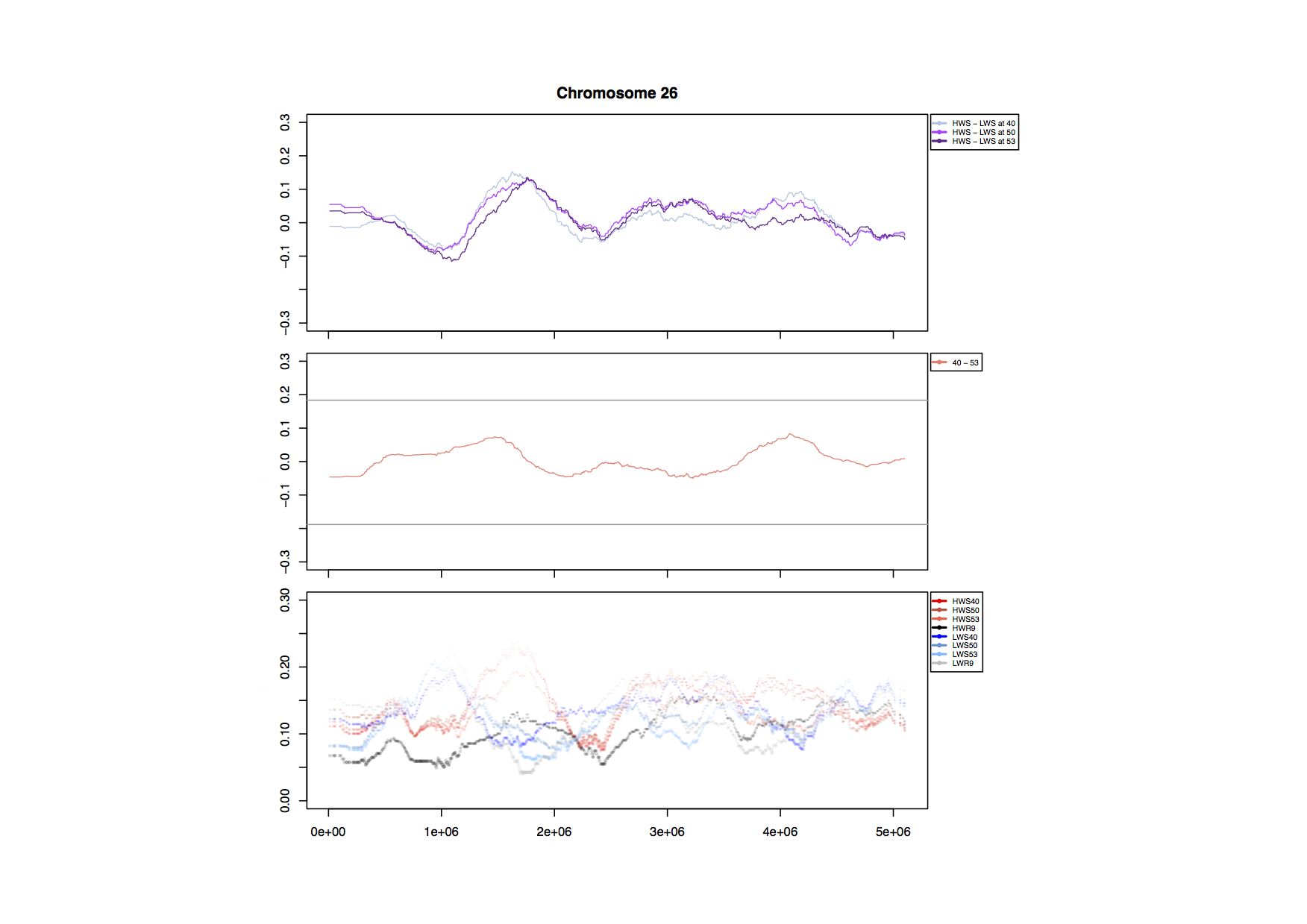

Supplement: Supporting Information [file supp_g3.113.008375_FileS3.zip › Figure_S1_chr26.tiff]

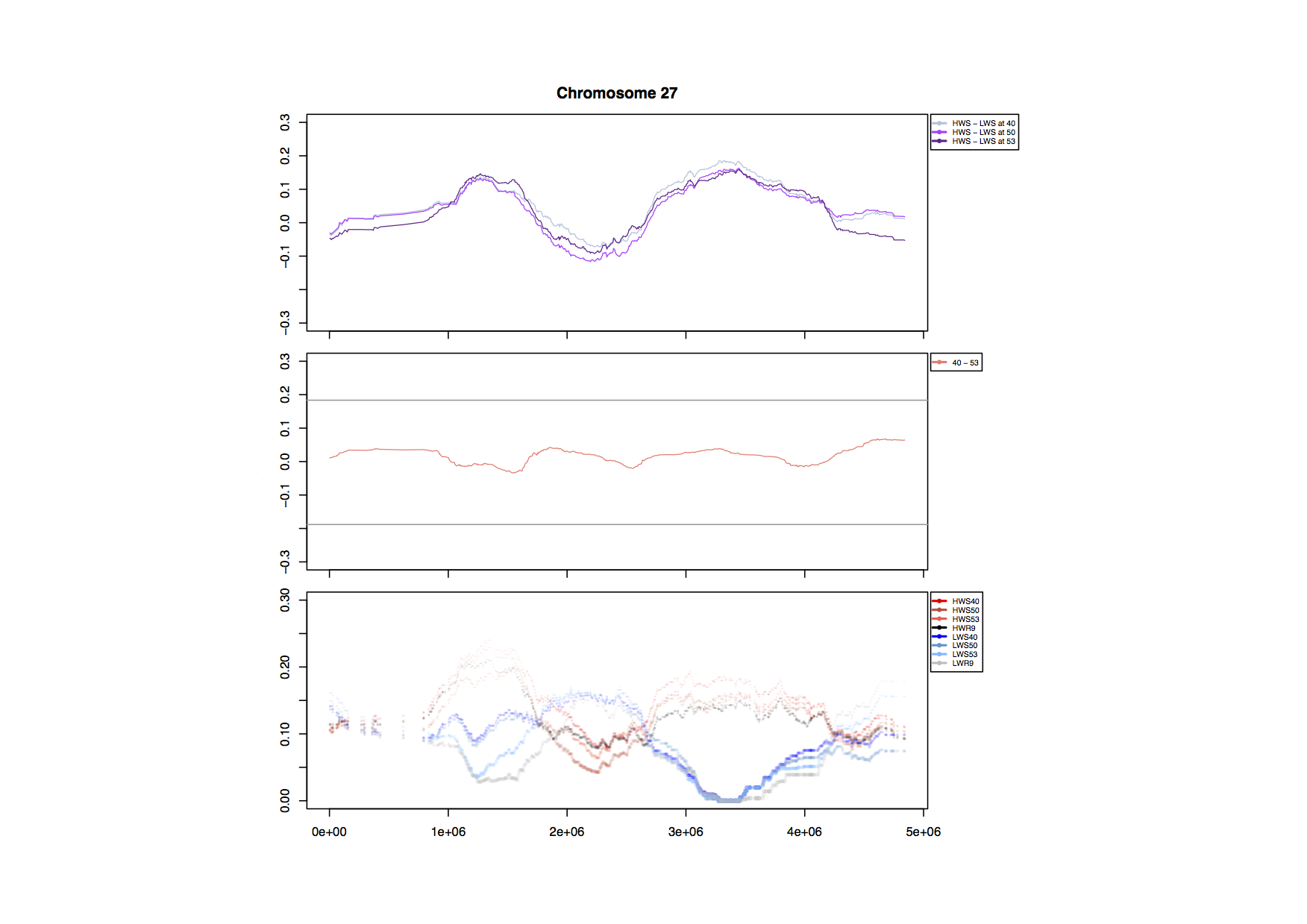

Supplement: Supporting Information [file supp_g3.113.008375_FileS3.zip › Figure_S1_chr27.tiff]

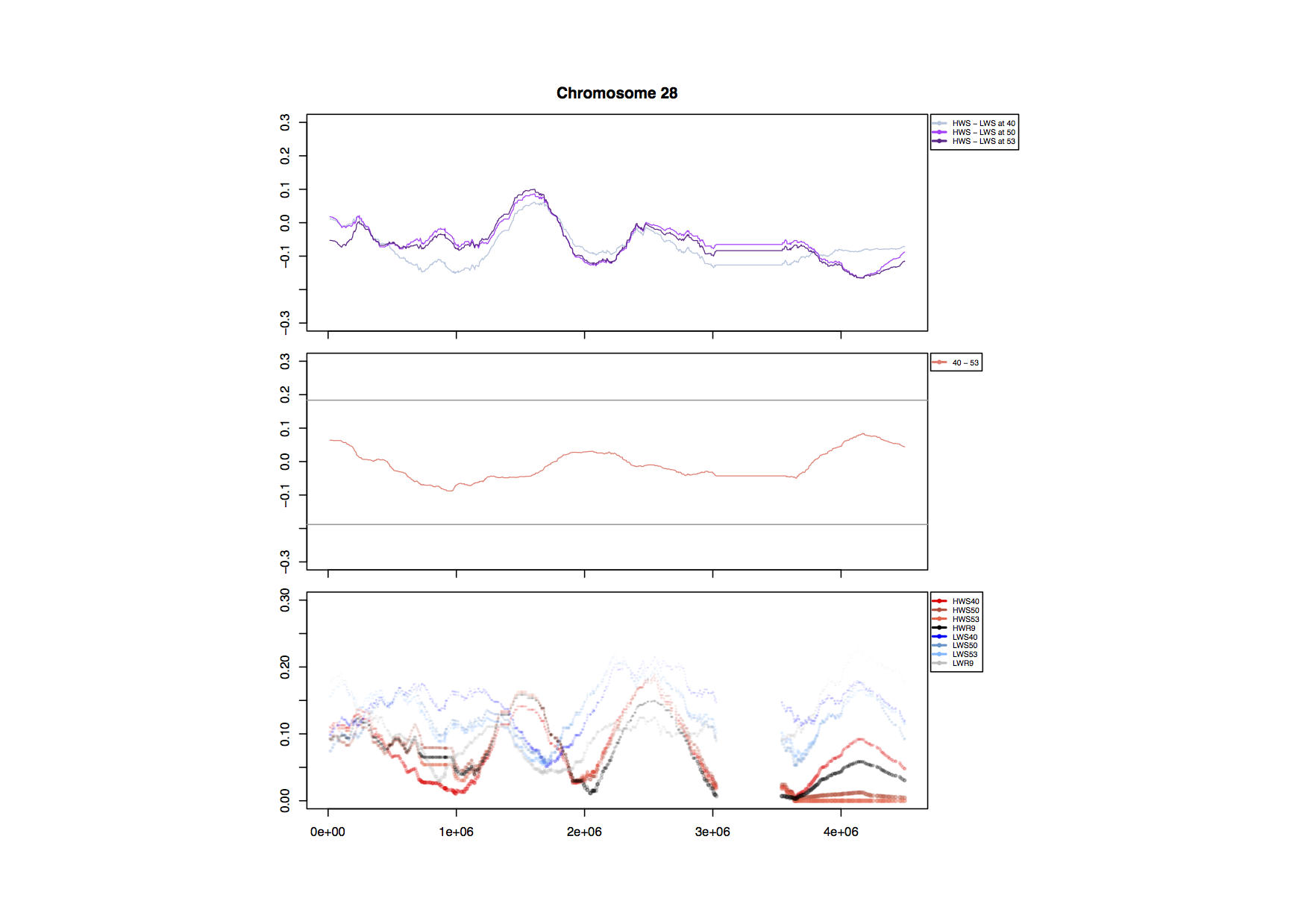

Supplement: Supporting Information [file supp_g3.113.008375_FileS3.zip › Figure_S1_chr28.tiff]

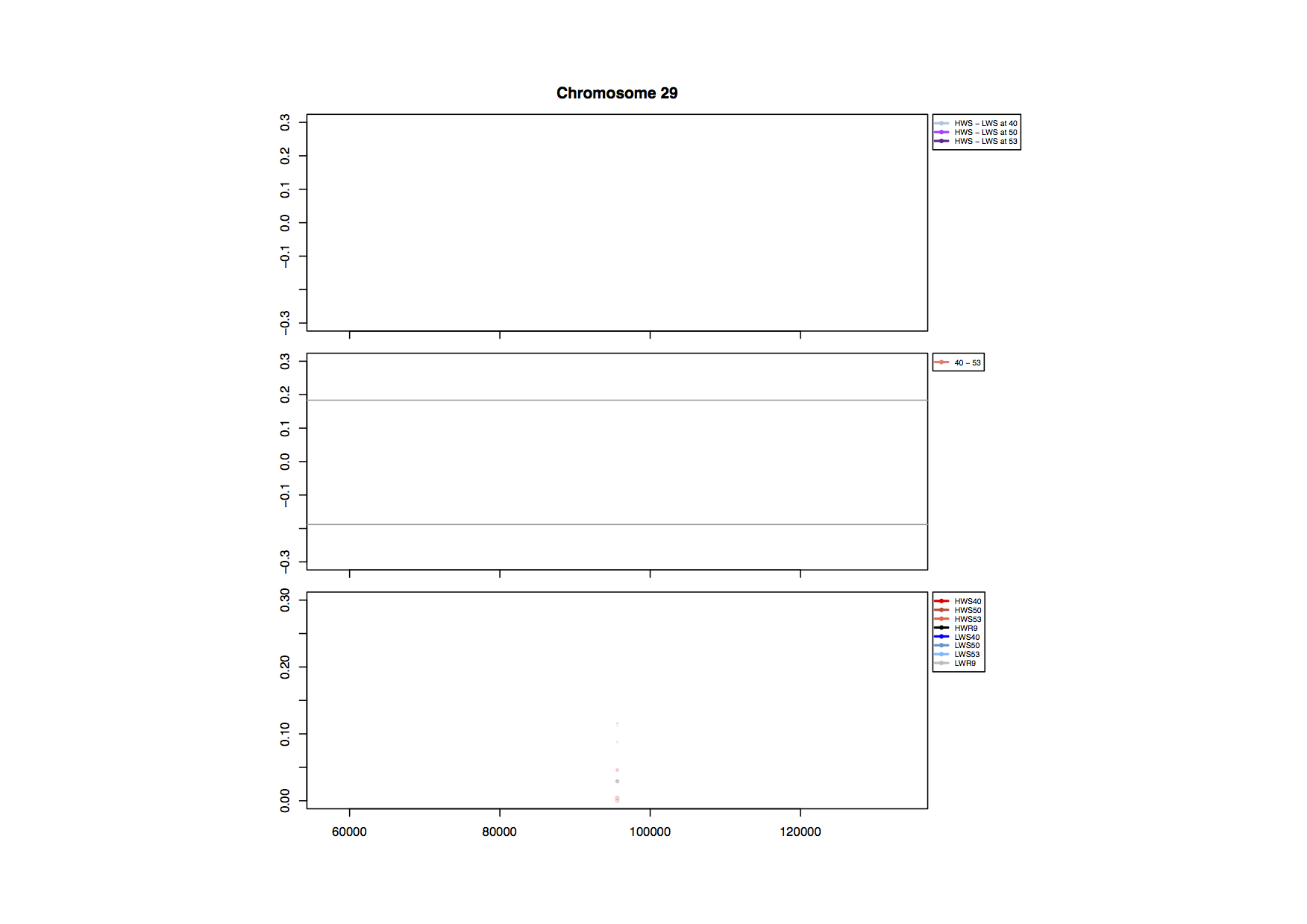

Supplement: Supporting Information [file supp_g3.113.008375_FileS3.zip › Figure_S1_chr29.tiff]

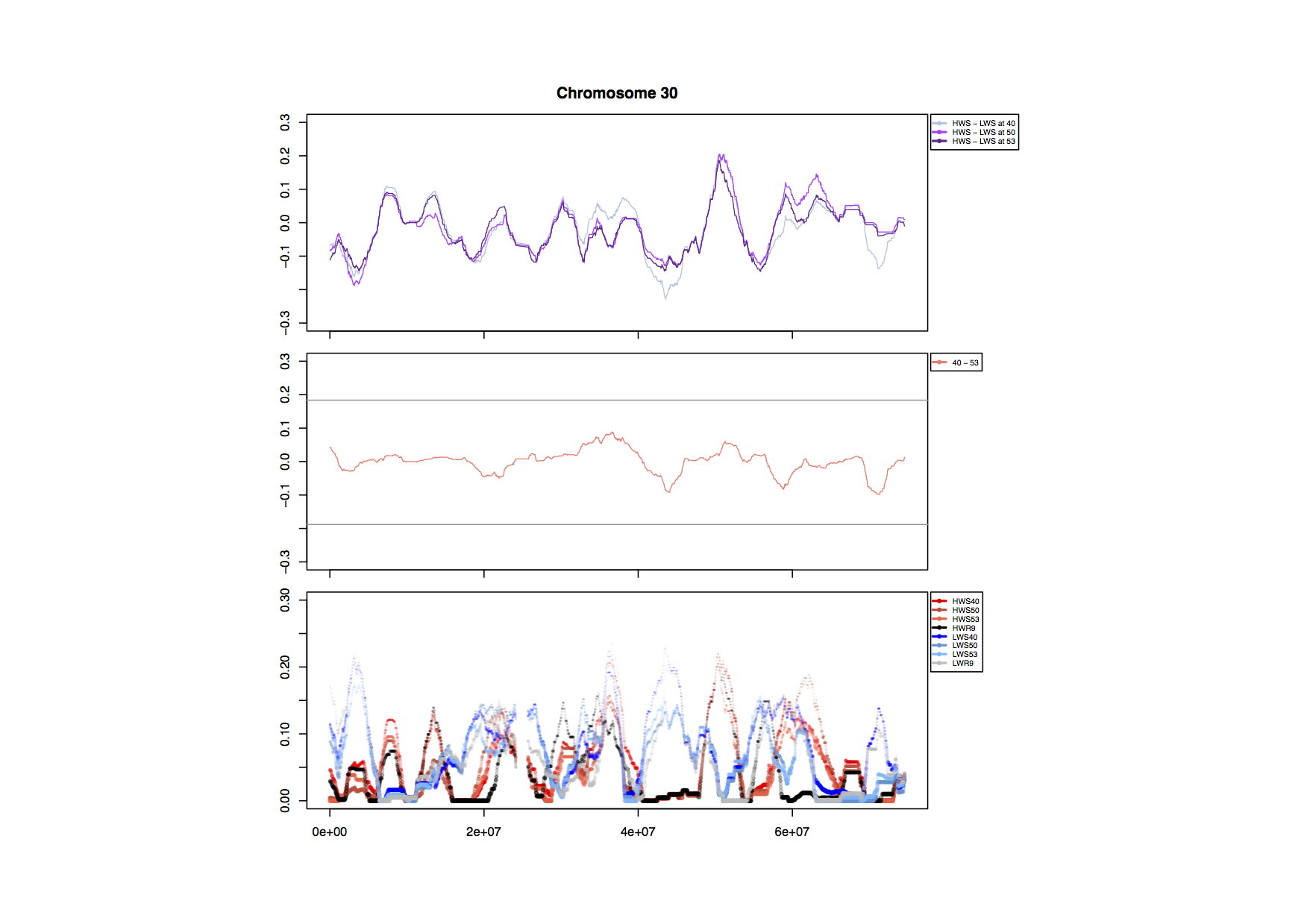

Supplement: Supporting Information [file supp_g3.113.008375_FileS3.zip › Figure_S1_chr30.tiff]

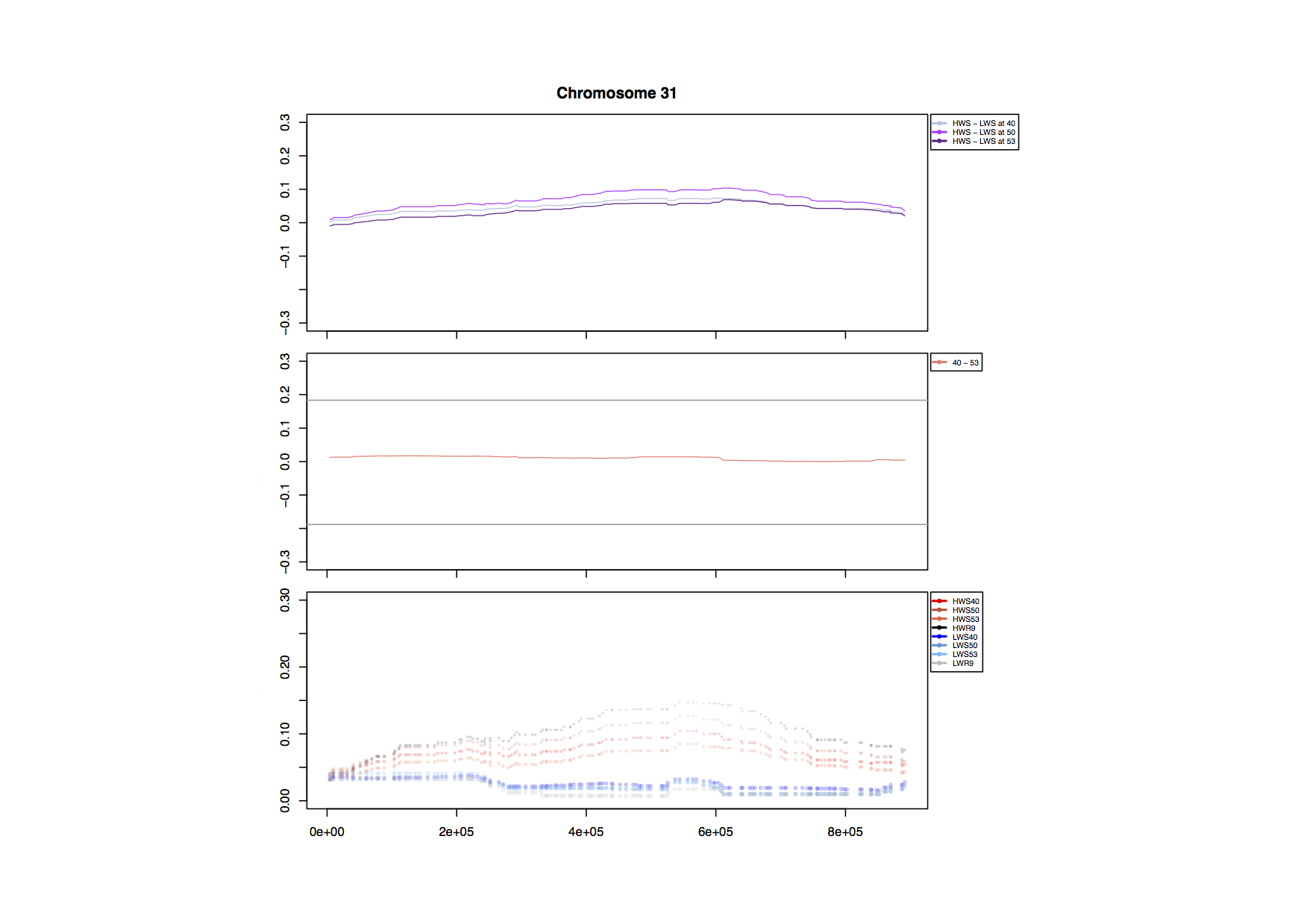

Supplement: Supporting Information [file supp_g3.113.008375_FileS3.zip › Figure_S1_chr31.tiff]

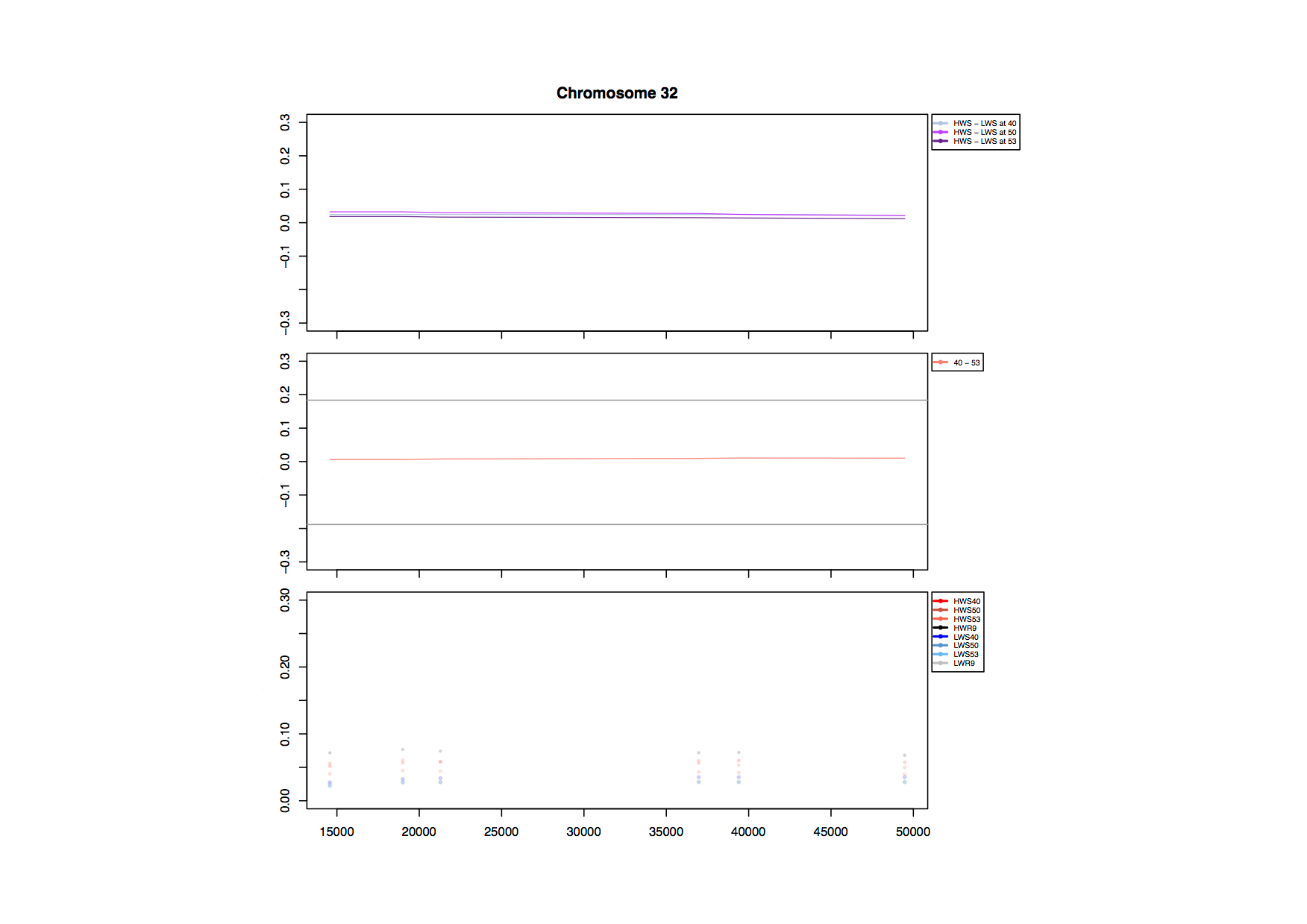

Supplement: Supporting Information [file supp_g3.113.008375_FileS3.zip › Figure_S1_chr32.tiff]
